# Patients’ User Experience of a Blended Face-to-Face and Web-Based Smoking Cessation Treatment: Qualitative Study

**DOI:** 10.2196/14550

**Published:** 2020-06-03

**Authors:** Lutz Siemer, Somaya Ben Allouch, Marcel E Pieterse, Marjolein Brusse-Keizer, Robbert Sanderman, Marloes G Postel

**Affiliations:** 1 Technology, Health & Care Research Group Saxion University of Applied Sciences Enschede Netherlands; 2 Centre for eHealth and Well-Being Research University of Twente Enschede Netherlands; 3 Digital Life Research Group Amsterdam University of Applied Science Amsterdam Netherlands; 4 Medical School Twente Medisch Spectrum Twente Enschede Netherlands; 5 Department of Health Psychology University Medical Center Groningen University of Groningen Groningen Netherlands; 6 Tactus Enschede Netherlands

**Keywords:** smoking cessation, cognitive therapy, blended treatment, smoking, user experience, tobacco, patient perspective

## Abstract

**Background:**

Blended web-based and face-to-face (F2F) treatment is a promising electronic health service because the strengths of one mode of delivery should compensate for the weaknesses of the other.

**Objective:**

The aim of this study was to explore this compensation by examining patients’ user experience (UX) in a blended smoking cessation treatment (BSCT) in routine care.

**Methods:**

Data on patients’ UX were collected through in-depth interviews (n=10) at an outpatient smoking cessation clinic in the Netherlands. A content analysis of the semantic domains was used to analyze patients’ UX. To describe the UX, the Hassenzahl UX model was applied, examining 4 of the 5 key elements of UX from a user’s perspective: (1) patients’ standards and expectations, (2) apparent character (pragmatic and hedonic attributes), (3) usage situation, and (4) consequences (appeal, emotions, and behavior).

**Results:**

BSCT appeared to be a mostly positively experienced service. Patients had a positive-pragmatic standard and neutral-open expectation toward BSCT at the treatment start. The pragmatic attributes of the F2F sessions were mostly perceived as positive, whereas the pragmatic attributes of the web sessions were perceived as both positive and negative. For the hedonic attributes, there seemed to be a difference between the F2F and web sessions. Specifically, the hedonic attributes of the web sessions were experienced as mostly negative, whereas those of the F2F sessions were experienced as mostly positive. For the usage situation, the physical and social contexts were experienced positively, whereas the task and technical contexts were experienced negatively. Nevertheless, the consequential appeal of BSCT was positive. However, the consequential emotions and behavior varied, ultimately resulting in diverse combinations of consequential appeal, emotions, and behavior (positive, negative, and mixed).

**Conclusions:**

This study provided insights into the UX of a blended treatment, and the results support the expectation that in a blended treatment, the strengths of one mode of delivery may compensate for the weaknesses of the other. However, in this certain setting, this is mainly achieved in only one way: F2F sessions compensated for the weaknesses of the web sessions. As a practical conclusion, this may mean that the web sessions, supported by the strengths of the F2F sessions, offer an interesting approach for further improving the blended treatment. Our theoretical findings reflect the relevance of the aspects of hedonism, such as fun, joy, or happiness in the UX, which were not mentioned in relation to the web sessions and were only scarcely mentioned in relation to the F2F sessions. Future research should further investigate the role of hedonistic aspects in a blended treatment and whether increased enjoyment of a blended treatment could increase treatment adherence and, ultimately, effectiveness.

## Introduction

### Blended Treatment

Health care is undergoing a sea change driven by the progress in digital technology [1]. One of the interesting innovations is blended treatment—a combination of the Web-based and face-to-face (F2F) therapy [2,3]. Blended treatment is a promising electronic health (eHealth) service because it is expected that the strengths of one mode of delivery will compensate for the weaknesses of the other [3-9]. For example, it is the strength of F2F treatment to be able to provide the personal attention of a professional that could compensate for the lack of F2F contact in Web-based treatment. In turn, one of the unique features of Web-based care is the accessibility, anytime and anywhere, which could compensate for the time in between F2F sessions when patients need support. Until now, there has been no final definition for blended treatment [3,6], and it is offered in various formats. The literature on blended treatment mentions different modes of delivery (eg, mainly Web-based [10,11], mainly F2F [12,13], 50-50 blend of Web-based and F2F [14]), different orders of F2F- and Web-based treatment (eg, sequential [10] or integrated [8,15]), and different tools for its use (such as platforms, emails, short message service, text messaging, and apps [5,16]). The intervention in this study is an integrated 50-50 blend of F2F treatment and treatment via a Web platform.

### User Experience and Blended Treatment

One of the main elements clarifying the individual’s use of services in general [17] and eHealth services, such as blended treatment, in particular [18], is the user experience (UX). UX refers to what people personally encounter, undergo, or live through while using, interacting with, or being confronted passively with systems [19]. Systems can denote products, services, and artifacts—separately or combined in one form or another—that a person can interact with [20].

Usually, the term UX refers to products, services, and objects that a person interacts with through a user interface [21]. However, for this study, we widened the scope of this term to explore the UX of a service (ie, blended treatment) that alternately uses computer-mediated communication *via* a user interface and F2F communication in counselling sessions.

Although a number of studies have examined the blended treatment [15], little is known about the patients’ UX specifically with blended treatments. An evaluation study (n=7) of a blended cognitive behavioral treatment for major depression [14] showed that while the patients’ pretreatment expectations were mainly neutral and some skeptical patients found it hard to start with the Web-based sessions, most patients appeared to have positive attitudes toward the blended treatment afterward. Another study [22] (n=14) on internet-based cognitive behavioral therapy for depression supported by short F2F consultations found that a sense of relatedness in terms of feeling connected to the therapist and being able to identify with the Web-based treatment may increase patients’ adherence to the blended treatment. Both the studies suggest that the elements of patients’ UX, such as expectations, usability, and identification, play a role in adherence to a blended treatment and should further be explored.

### Patients’ User Experience

**Figure 1:**
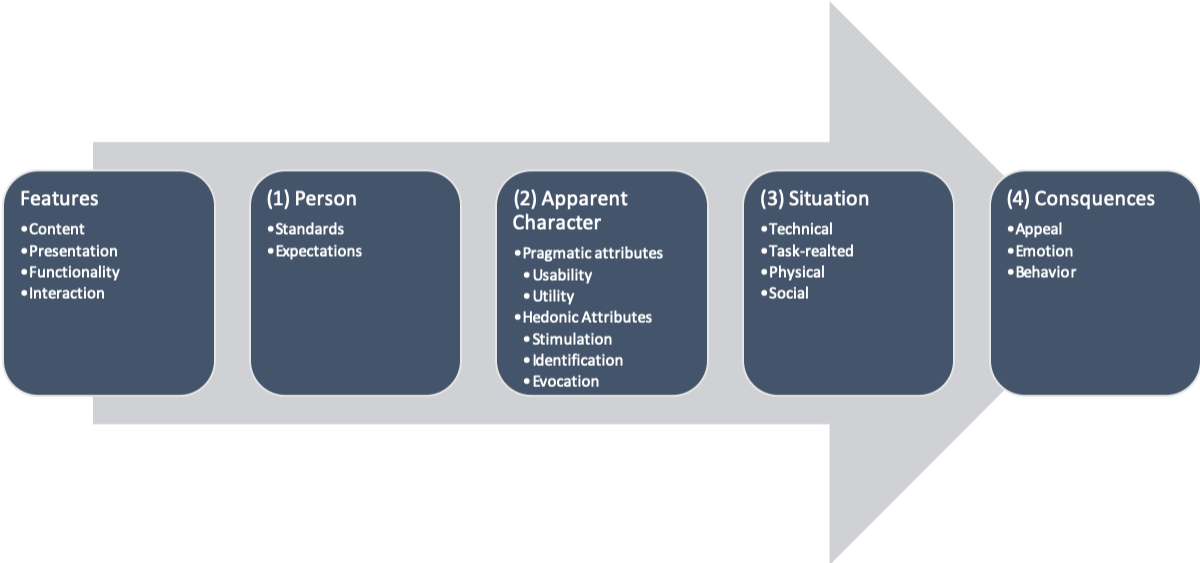
Key elements of the user experience model.

For the patients’ perspective on the blended care treatment, Hassenzahl’s model of UX from a user’s perspective was adapted [21,23-25]. This process-oriented constructivist model defines five key elements and their functional relations (Figure 1). Basically, the model states that while getting in contact with the *features* of a product or service, a process is triggered, in which the user constructs the UX (this is illustrated by the grey arrow in Figure 1). In the beginning, the user constructs—moderated by the person’s *standards and expectations*—an *apparent character* of the product or service. Moderated by the specific usage *situation,* the apparent character will then finally mediate a number of *consequences.*

The features of the service (in this case the blended treatment) are selected and combined by the treatment developers independently of the patients that ultimately follow the treatment. Since the features are not constructed by the users, the product features only play a minor role in this study. In turn, the focus is placed on the patients’ response to the treatment’s features to explore the UX from the user’s perspective in a narrower sense. This means that the UX from a user’s perspective is built based on only four of these five key elements: (1) the patient’s standards and expectations, (2) the apparent character, (3) the usage situation, and (4) the consequences. In the following paragraphs, each key element is described and illustrated by examples of how it applies to the blended treatment in this study.

### Features

The *features* of a product or service refer to its *content*, *presentation*, *functionality*, and *interaction* [23,24]. The content of the treatment of this study—Blended Smoking Cessation Treatment (BSCT)—refers, for example, to the behavioral change techniques for smoking cessation [26] that comprises BSCT. The presentation refers to the clinical surrounding as BSCT is part of the routine care setting of a hospital. Functionality and interaction refer to the F2F and Web-based sessions, which offer synchronous interactions with the counselor (eg, functions, such as providing feedback on behavior and building rapport) and asynchronous counselor-independent interactions with the Web-based system (eg, functions, such as self-recording of smoking behavior *via* a Web-based smoking diary). More details about the study intervention are provided below in the Methods section.

#### Person

The patients’ *standards* and *expectations* are based on their experiences with the other services [23,24] with which the patient can compare BSCT. If a patient compares BSCT with, for example, earlier experiences in health care, smoking cessation support, F2F treatment, or use of computers and internet, the patient may start BSCT with a subjective standard, such as “using the computer for treatment is too difficult for me,” or with an expectation, such as “blended treatment will be more comfortable because I can partly do treatment at home.”

#### Apparent Character

When confronted with a service, an apparent character is constructed by the user. The apparent character is a cognitive structure representing *pragmatic* and *hedonic* attributes [23,24]. Pragmatic attributes refer to the *utility* (eg, “supporting,” “useful”) and *usability* (eg, “clear” and “easy to use”) of a service, such as BSCT. Hedonic attributes of BSCT refer to *stimulation* (eg, “novel and interesting” and “makes me think”), *identification* (eg, “my style”), and *evocation* (eg, “reminds me of filling in tax forms”).

#### Situation

The usage situation moderates the consequences of the apparent character [23,24] and refers to the *technical*, *task-related*, *physical*, and *social* contexts. These situations are different between patients and over the course of the treatment, especially for the Web-based sessions. For example, filling in a smoking diary while being on your own in a silent surrounding may result in different consequences than doing this in the living room with a partner and children around you.

#### Consequences

The fit of the apparent character and the usage situation leads to three consequences: *appeal*, *emotions*, and *behavior* [23,24]. For patients, BSCT, for example, may appeal as “fine” while feeling “satisfied” and “adhering to the treatment.”

### Aims of This Study

As UX has been shown as an important factor in explaining the behavior of a user in general [27], and patients’ use of health care services in particular [28], the aim of this study is—from a UX point of view – to explore whether in blended treatment the strength of one mode of delivery may compensate for the weaknesses of the other. By applying Hassenzahl’s model of UX to qualitatively describe the patients’ UX of BSCT in routine care, the question what positive and negative experiences patients have with BSCT in general and with the F2F sessions and the Web sessions in particular will be addressed. This research will contribute to a deeper understanding of the facilitators and barriers to blended treatment, which will provide new insights for both scientific research on blended treatment and its improvement in clinical practice. It is expected that the application of the findings on UX elements in furthering the development of blended treatment will lead to better treatment outcomes.

## Methods

### Study Intervention

BSCT is a clinician-led intervention [1] which combines F2F and Web-based treatment delivered in routine care settings at the Outpatient Smoking Cessation Clinic (Stoppen met Roken Poli [SRP]) of the Department of Pulmonary Medicine at Medisch Spectrum Twente Hospital in Enschede, The Netherlands. BSCT is derived from the Dutch Guideline Tobacco Addiction [29], fulfilling the requirements of the Dutch care module for smoking cessation [30]. The treatment is based on both the F2F treatment as usual at SRP [31,32] and Web-based treatment at Tactus Addiction Treatment (www.rokendebaas.nl). A team of clinical experts from both the organizations developed BSCT, striving for a 50-50 mix with constant alternating of F2F and Web-based treatments by replacing five of the usual ten F2F sessions with appropriate Web-based sessions. This treatment design decision was made based on the randomized controlled trial (LiveSmokefree study [8]), which compared the effectiveness of BSCT with F2F treatment. The order, planning, mode of delivery, and main content of the BSCT sessions is shown in Table 1. The details of BSCT have been described in earlier papers [8,15]). To provide an impression of the look and feel of the Web interventions, Multimedia Appendix 1 shows screenshots of the Web sessions of BSCT.

**Table 1:** Order, planning, mode of delivery, and main content of the blended smoking cessation treatment sessions.

Session	Week	Mode of delivery	Content
1	1	Face-to-face	Goal setting
2	3	Web-based	Measures for self-control
3	5	Face-to-face	Dealing with withdrawal
4	7	Web-based	Breaking habits
5	9	Face-to-face	Dealing with triggers
6	11	Web-based	Food for thought
7	14	Face-to-face	Think differently
8	18	Web-based	Do differently
9	22	Face-to-face	Action plan
10	26	Web-based	Closure

### Setting and Participants

The current study is a substudy of the LiveSmokefree study—a single-center randomized controlled noninferiority trial with parallel group design, which examines the effectiveness of BSCT as compared with F2F treatment. The inclusion criteria for the LiveSmokefree study were (1) aged 18 years or older, (2) willing to quit smoking, (3) current daily smoker (at least one cigarette a day), and (4) speaking/reading/writing Dutch.

A purposive sample (n=10) of the participants from the blended arm of the LiveSmokefree study [8] that had already ended the treatment was selected, striving for a heterogeneous mix of patients regarding the characteristics (Table 2) that were expected to influence the patients’ UX (ie, age, sex, educational level, adherence, counselling, and quitting success). For recruitment, the patients were called and invited by the research assistants to participate in the UX study. The participation was voluntary; patients had to sign an informed consent form and received no incentives.

**Table 2:** Purposive sample.

Characteristics	Randomization number									
	10	12	14	25	27	34	53	75	106	509
Age (years)	77	54	68	71	37	45	60	65	37	58
Sex (m: male; f: female)	m	m	m	f	f	m	m	f	m	m
Education level	Low^a^	Mid/high^b^	Mid/high	Low	Low	Mid/high	Low	Mid/high	Low	Mid/high
Internet skills^c^	28	34	37	38	38	46	36	40	40	39
Nicotine dependence^d^	5	7	4	6	7	4	3	2	7	6
#Adherence F2F^e^	3	4	6	5	2	3	5	2	8	2
#Adherence Web^f^	3	2	8	2	0	3	6	9	7	2
#Adherence BSCT^g^	6	6	14	7	2	6	11	11	15	4
Adherence F2F^h^	N	N	Y	Y	N	N	Y	N	Y	N
Adherence Web^i^	N	N	Y	N	N	N	Y	Y	Y	N
Adherence BSCT^j^	N	N	Y	N	N	N	Y	N	Y	N
Counselor^k^	A	B	B	B	B	A	A	C	C	B
Stopped smoking^l^	Yes	No	Yes	No	No	No	Yes	No	Yes	No

^a^Low: lower than vocational education and training.

^b^Mid/high: vocational education and training or higher.

^c^Internet skills: range 10-60; higher number indicates better skills.

^d^Nicotine dependence: Fagerström, range 0-10; higher numbers indicate higher nicotine dependency.

^e^#Adherence F2F: adherence to face-to-face (F2F) sessions, range 0-8, based on the 8 activities belonging to F2F sessions; higher number indicates higher adherence.

^f^#Adherence Web: adherence to Web sessions, range 0-10, based on the 10 activities belonging to Web sessions; higher number indicates higher adherence.

^g^Adherence BSCT: adherence to blended smoking cessation treatment (BSCT) in general, sum of #Adherence F2F and #Adherence Web, range 0-18; higher number indicates higher adherence.

^h^Adherence F2F: categorical classification of adherence to the F2F sessions based on a 60% threshold (Y= adherent; N=nonadherent).

^i^Adherence Web: categorical classification of adherence to the Web sessions based on a 60% threshold (Y= adherent; N=nonadherent).

^j^Adherence BSCT: categorical classification of adherence to BSCT in general based on a 60% threshold (Y= adherent; N=nonadherent).

^k^Counselor: who carried out the treatment.

^l^Stopped smoking: self-reported abstinence.

### Ethics

Both the LiveSmokefree study and this substudy on patients’ UX were approved by the accredited Medical Research Ethics Committee Twente (P14-37/NL50944.044.14). The LiveSmokefree study was registered in the Dutch Trial Registration (NTR5113).

### Data Collection

Qualitative data about the patients’ UX was collected by in-depth semi-structured interviews. The interview guide (Multimedia Appendix 2) was developed following the key elements of the UX [23,24] to elicit both the patients’ standards and expectations toward BSCT, the apparent character of BSCT (usability, utility, stimulation, identification, and evocation), the usage situation (technical, tasks, physical, and social), and the consequences (appeal, emotions, and behavior). Additional interview questions were created from a clinical perspective addressing practicalities (eg, intake procedure, treatment procedure, and adherence) and ideas for the improvement of current BSCT.

The interviews were conducted by the first author (LS) between October 2016 and March 2017. Because LS is not a Dutch native speaker, he was supported by trained Dutch research assistants to avoid possible ambiguities and linguistic misunderstandings. On the date of the interview, the interviewees were picked up from the waiting area of the SRP and led to a neutral meeting room. After receiving permission for audio recording, the interviewer read a written introduction, which emphasized that the patient was invited to recall and describe (“tell stories”) their UX. After this briefing, a general stimulus (“Can you, first of all, tell us what your experiences with the blended treatment are? We would like to hear all the events and experiences that were important to you.”) was used to start. Interviews followed a detailed written interview guide (Multimedia Appendix 2), but were open-ended in nature, allowing the interviewers to ask probing questions and to follow up on interesting topics and experiences related to BSCT.

The audio-recordings were transcribed verbatim by trained research assistants following the guidelines for data preparation and transcription, as described by McLellan et al [33], and were subsequently analyzed using the qualitative data analysis software ATLAS.ti Version 8.3.1 (ATLAS.ti Scientific Software Development GmbH).

### Auxiliary Data

The data regarding the patients’ age, sex, education level, internet skills, nicotine dependence, and counselor (Table 2) were acquired from the LiveSmokefree study database, for which the data were collected using a Web-based questionnaire that the patients completed at the beginning of the treatment. A detailed description of the variables and their measurements can be found in the protocol article of the LiveSmokefree study [8]. The patients’ characteristics were reported as medians with IQRs or as numbers using SPSS version 24.

The data about adherence and smoking status (Table 2) were acquired from a dataset build in 2018 for a paper on adherence to BSCT [15]. Based on 18 patient activities that reflect the course of the treatment (eg, attending a F2F session or completing a Web-based task), an adherence score ranging from 0 (nonadherent to any activity after the first treatment session) to 18 (adherent to all activities) was calculated for each patient. The patients’ adherence rates were reported as medians with IQR using SPSS version 24. Based on a 60% threshold for both the F2F sessions and the Web sessions [15], the patients were classified as adherent or nonadherent to BSCT. Questions regarding adherence were also asked in the interviews (see above), which might have led to different assessments (eg, patient #25). To examine the self-reported smoking status (stopped smoking: Yes/No), data from both the in-depth interviews and the follow-up Web-based-questionnaires of the LiveSmokefree study 6-month after the treatment start were used. In case the interview and questionnaire data contradicted each other, the interview data were considered superior.

### Codebook Development

Based on the semi-structured interview guide, content analysis was used to analyze all the interviews. The codebook was developed by two research team members (LS, SA), building on the interview guide and the research goals related to the clinical setting (eg, ideas for improvement of BSCT) [34]). The codes were grouped in semantic domains and intercoder agreement was analyzed per semantic domain using the intercoder analysis feature of Atlas.ti 8.2.4. The disagreements were discussed, and the codebook was revised until acceptable agreement (Krippendorff c-α-binary 0.650-0.928) for each semantic domain was achieved. The codes, their description, and the intercoder agreement per semantic domain are displayed in Multimedia Appendix 3.

### Paraphrasing and Regrouping

After coding, all coded Dutch quotes were paraphrased in English by LS and collected in a table (Multimedia Appendix 4). Applying Hassenzahl’s model of UX from a user’s perspective [23,24], the semantic domains of the codes were revised by linking the codes to the four of the five key elements, which form the UX from a user’s perspective: (1) patients’ standards and expectations; (2) apparent character (pragmatic attributes: usability, utility; hedonic attributes: stimulation, identification, and evocation); (3) usage situation (physical, social, technical, task); and (4) consequences (appeal, emotion, behavior). Finally, the UX was described for each key element distinguishing as far as possible between BSCT in general (ie, the experience of BSCT as a whole) from the two modes of delivery (ie, the F2F sessions and the Web sessions). Furthermore, in describing the UX, an attempt was made to make a distinction between the positive and negative UX, which is based on the idea that UX is a “primarily evaluative feeling (good/bad) while interacting with a product or service” [25]. Ultimately, we summarized the variety of consequences in three kinds of combinations of consequential appeals, emotions, and behavior.

## Results

### Overview

In the following, the patient characteristics are presented first. Then, the positive and negative statements for each key element are described. As far as possible, this is done first for BSCT in general and then for the F2F and Web sessions. It is to be noted that the analysis and presentation methods were clarified after the interview phase, and the statements were not always available in every area.

### Participants

Patients’ characteristics are shown in Table 2. The median age of the patients was 59.0 years (IQR 43.0-68.8), and the majority were males (7/10). Half (5/10) of the patients’ educational level was lower than vocational education and training. The median internet skill level (range 10-50, higher numbers indicate higher skills [8]) was 38.0 (IQR 35.5-40.0), and the median nicotine dependence (Fagerström range: 0-10, higher numbers indicate higher dependency [35]) was 5.5 (IQR 3.8-7.0).

### Patients’ Standards and Expectations

In general, the patients approached BSCT mostly with a positive-pragmatic standard and a neutral-open expectation. None of the patients had followed a blended treatment or a Web-based treatment before. Therefore, their standards and expectations were based mainly on earlier experiences with F2F sessions, with earlier stop smoking attempts, and with ICT use in general. Only one patient (#34) used health-apps (Mindfulness, Stoptober). However, most of the patients (7/10) had received F2F counseling before, participated in a group therapy (#34), or were familiar with mindfulness (#34, #53).

For *F2F sessions*, positive standards predominated. Patients said, for example, that “Human touch is important” (#75), quitting is easier with F2F support (“with help stopping will be easier” [#53, #14]), F2F treatment is “ideal,” and it “adapts to your competencies” (#12). One patient, however, considered that it “can be hard if you dislike the counselor” (#14).

Building amongst others on *earlier stop smoking attempts*, patients had the standard that the quitting success may depend on themselves (reporting, eg, “Stopping you have to do for yourself.” [#12]; “Treatment only makes sense if you have the will to stop” [#14]; quitting is “more a mental than a physical problem” [#27]; “You have to be strong” [#27]; or “You just have to do the things” [#53]), on missing support (“With help stopping will be easier” [#53, #14], and on stress (“Relapses are caused by stress” [#10, #34, #75]).

For *ICT-use in general*, while being familiar with using ICT (eg, searching the Web, using email/WhatsApp), the majority of patients showed a pragmatic standard “Computer is a tool” (#509, #75); “I am not a computer freak” (#12); “Computer is not my way” (#10); or “I am neither a forerunner nor a left behind” (#25). Only one patient (#34) reported that he “personalizes his mobile.” Most patients also emphasized that they do not prefer computer-mediated communication over F2F communication because it “leads to misunderstandings” (#53), “it is easier to cheat online” (#34, #12), “it is easier to do sloppy” (34), “online information is not as important as written on paper” (#25), or “I do not trust internet information” (#509). Three of the patients (#106, #27, #25) reported that they use mobile devices (smartphone, tablet) more often, for example, “I use the laptop less since I have a tablet” (#25) or “I prefer mobile over PC” (#27).

Referring to *BSCT in general*, most patients (#106, #34, #27, #75, #25) described their *expectations* as “neutral” or “not clear,” while some (#53. #34, #10) emphasized to expect support from BSCT, saying, for example, that they want the counselor to be “a driving force” (#10) or that they expect “to get more grip on smoking cessation” (#34). One patient (#14) remarked that BSCT “is new and sounds interesting.”

### Apparent Character of Blended Smoking Cessation Treatment

While being confronted with BSCT and moderated by their standards and expectations, the apparent character of BSCT that the patients constructed, seemed to be both positive and negative. The *pragmatic attributes* (*usability* and *utility*) were experienced mostly positive while the *hedonic attributes* (*stimulation*, *identification*, and *evocation*), especially for the Web sessions, tended to be negative.

#### Pragmatic Attributes of Blended Smoking Cessation Treatment

BSCT’s pragmatic attributes (*usability* and *utility*) were experienced as good. However, some patients also criticized pragmatic aspects of BSCT, especially of the Web sessions, which indicated possibilities for further improvements.

#### Usability

Most patients experienced the *usability of BSCT in general* as positive, reporting, for example, that the “intake was good” (#75, #10 ,#14), “there have been no problems” (#509), “everyone was kind” (#34) “everything was clear and easy to use” (#53), “all was quite logical” (#14), the “treatment was picked up well” (#53, #27, #10), “BSCT parts connected to each other” (#106, #75, #14), and that “the intervals between sessions were fine” (#53). One patient (#14) reported “less travelling” (Note: BSCT patients only had to attend 5 F2F sessions at the clinic, compared with 10 F2F sessions in the F2F treatment as usual) as an advantage of BSCT, while another patient (#10) found that “still having to travel to the hospital at all” is a disadvantage. Further negative aspects of usability reported were the “long waiting list” before treatment start (#14, #12) (Note: regular waiting time before treatment start was around two months), “the long waiting times” in the waiting area before start of a F2F session (#14), that it was “not clear where to turn to outside the office hours” (#14), that “intervals between sessions were too long” (#10, #25), and that “not everything was explained in detail” (#25) and that the patient was “surprised about the order of the sessions” (#25).

The *usability of the F2F sessions* was experienced as “easy” (#25) or as “easier than web” (#27). Yet some patients criticized “that the counselor did not have enough time” (#12, #10, #14) or that the sessions were “slow and time consuming” (#27, #14).

Six patients (#509, #106, #53, #27, #75, #14), experienced the *usability of* *Web sessions* as “easy to use,” while three patients (#34, #10, #25) reported the opposite (“not easy to use”). The patients criticized that the Web sessions were “too time demanding” (#509, #106), there was “a lot of repetition” (#10, #53, #27, #14), they “did not get immediate response” (#14), they “did not receive online assignments” (#27), and the “login would have been easier if you do not have to remember your password” (#27). Furthermore, two patients (#509, #106) reported that they did the smoking registration on paper before doing it on the Web because “it was simpler” (#106). However, this was “double work” (#106). Yet the patients liked “to be notified about new Web content automatically” (#75), that “emails and phone calls raised awareness” (#34), that “filling in forms online was handy” (#34), and “online saved time” (#25).

#### Utility

With regard to the *utility of BSCT in general*, the patients experienced the utility as positive, finding that “all BSCT parts were helpful—some more, some less” (#53), BSCT “matched my quitting process” (#53), “all has been discussed” (#106), “there was progress” (#106), BSCT “offered support” (#27), or “Web only would not have offered what I needed” (#75).

The *utility of the F2F session* was experienced as positive by most patients (7/10) also. Patients reported that F2F “offered flexibility” (#75) as “I could talk to the counselors about all of my problems” (#509), “all has been discussed” (#14), that with F2F “it was easier to ask questions” (#14), F2F “you got direct answers” (#14), F2F “stimulated more than web” (#10), and F2F “with medication was better than medication only” (#106). The counselors “reinforced” (#53, #25), “stimulated” (#53, #14, #10), “offered support” (#53, #25, #14), “shared good metaphors” (#53), and “explained everything very well” (14). Three patients experienced the F2F session as not useful, saying that the counselors “did not offer enough support” (#34, #27, 12), “did not reinforce” (#12), “did not motivate” (#12), “did not discuss all alternatives” (#34), and “asked too much questions” (#27).

For the *utility of the Web sessions, there were both positive and negative experiences*. Some patients had a predominantly negative experience saying that “reporting *via* Web was too time demanding” (#509), that Web “offered too much information” (#106), that Web “did not match my quitting process” (#27, #14), that “a computer does not answer” (#14) and that Web “does not work for me” (#75). Furthermore, ideas for improvement were reported, such as “an App would be better than web” (#34) and other services should be included, such as “short reinforcements *via* WhatsApp, emails, in-between sessions, video instructions, helpdesk, chat support, short instructions” (#34) and “audio information” (text to speech) (#27). However, patients also reported positive experiences saying that the Web “offered support in difficult moments” (#53), Web “offered tips” (#53), and “it was good to have information available online” (#27, #14, #34).

#### Hedonic Attributes of Blended Smoking Cessation Treatment

For the hedonic attributes (*stimulation*, *identification*, and *evocation*), BSCT was experienced both positively and negatively. While some patients felt *stimulated* by BSCT, others reported being demotivated. Especially for the Web sessions, most patients reported low *identification*. Also, the Web sessions *evoked* mostly negative comparisons and induced several ideas for improvements.

#### Stimulation

Patients reported both positive and negative stimulation by *BSCT in general* and rather low stimulation referring to the *F2F sessions* and *Web sessions*.

For *BSCT in general*, patients—on the one hand—felt stimulated to “quit smoking” (#14), to “discuss costs of smoking” (#12), to “think” (#106, #34), to “dig deeper” (#509), or to “look back” (#75). Patients also reported that the carbon monoxide measurements during the F2F sessions stimulated quitting (#53, #12). On the other hand, patients reported that “BSCT did not offer new things” (#34) or was “not interesting” (#14), and that certain interventions (ie, dealing with tempters) were “not new” (#25). Furthermore, patients were demotivated by “always the same questions” (#27), by “digging too deep” (#27), and by contradictory goals (quitting smoking vs weight reduction) (#27).

For the *F2F sessions*, patients said that the “counselor had no impact” (#27, #12, #14, #25). However, some patients (#12, #509, #34) reported that they were reinforced by the counselors to use the Web sessions.

For the *Web sessions*, one patient said, that Web “broadened your awareness” (#75), whereas the majority of patients reported no or low stimulation saying that “online won’t get through to me” (#53, #34, #14, #25), “online exchange with the counselor did not affect extraordinary” (#25, #509), and to be demotivated by the Web sessions (#10, #509) or computer use (#106).

#### Identification

For *BSCT in general* patients could identify linking to individual features, such as “perseverance” or “self-control.” However, for the Web sessions, most patients reported low identification. The ones showing higher identification with the Web sessions did this by referring to personal contact with the counselor. Patients found it easier with the *F2F sessions* than the *Web sessions* of BSCT.

Related to *BSCT in general,* patients reported that BSCT linked to individual features, such as “perseverance” (#75), “self-control” (#75), “the ability to work based on reading and writing” (#75), “IT-skills” (#10), and “age” (#10). However, one patient (#27) reported that she “felt treated like a child” and that she “lost her rhythms.”

For the *F2F sessions* patients reported that these “felt more familiar” (#106), that patients liked “the F2F sessions the most” (#53) and “talking to the ladies” (#10) (Note: by this the male patient (#10) refers to the female counselors).

For the *Web sessions,* most patients reported low identification, saying, “I don't feel like it much” (#106) or “not to like online” (#106, #10), that “online is not my style” (#12, #75, #10, #25), to “prefer on paper” (#25), or being “too stupid for IT” (#10). One patient (#75) showed a higher identification with the Web sessions, emphasizing “Web I did for myself,” “I know why I did Web,” and “I understood the process.” In turn, she criticized saying that “online did not give the opportunity to make it more personal” (#75). Three patients reported that the Web parts supported their personal contact with the counselor, mentioning that *via* Web parts “I had contact with her” and “they knew something about me” (#509), that “during the F2F sessions it became clear that the counselor reads the Web content“ (#25), that “I had the idea that it is used on the other side” (#53), and that “you knew there is someone behind it” (#34). In turn, three patients reported that “you didn’t know who has written the content” (#15), that “computer did not talk to you” (#12, #14), and that “you did not get the feeling that there is a human being on the other side” (#12).

#### Evocation

For the *Web sessions,* the patients reported several negative comparisons, such as “Web was like handling a machine, because you are not sitting opposite to each other” (#106), Web sessions were like “bookkeeping” (#53, #34, #14), like “a manual” (#53), like “filling in tax forms” (#10), and like “paper” (#27).

### Situation

For the usage situation, mostly the *technical* context had a negative impact on the UX. Especially the *Web sessions* depended on the technical factors, which were criticized. Furthermore, referring to the *task* context of *BSCT in general*, some patients reported not having enough time for the treatment. Both the *physical* and the *social* context were described as mostly positive.

#### Technical

For the technical situation, the patients referred to the *Web sessions*, criticizing by saying that Web “did not work on iPad” (#34, #10, #25, #75). Although the patients had been informed at start of the treatment that the software for the Web session could not be used on tablet computers, they would have preferred to use tablets because the “Tablet is always on, Laptop not” (#34, #75, #14, #25) and tablet “is more comfortable” (#10), or because they (#10, #25) moved from laptop to tablet during BSCT. Furthermore, for the use of computers for the Web sessions, the patients criticized by saying that they “had to start up the laptop, which takes time” (#106, #34, #14).

#### Task

Referring to tasks, patients reported not to have enough time for the BSCT “because of other tasks” (#509) or “because of family tasks” (#106), or to feel “sometimes stressed—sometimes relaxed” (#27).

#### Physical

For the *F2F sessions*, the patients reported little about the physical usage situation, mentioning only “that I live close to the hospital” (#25) and “that the treatment took place in the old building, which was not a nice place” (#34, #27) (Note: Between the patients treatment and the interviews the department moved to a new building).

For the *Web sessions*, the patients shared more information about the physical usage situation reporting that they did the Web sessions at “my own home office” (#25, #509), in a “hobby room upstairs, which is a nice place” (#10), “upstairs, where it is quite hot in the summer” (#14), “with the laptop at the dining table with wife and children around me” (#53), “in the kitchen” (#106), and “with laptop lying on the bed in the sleeping room” (#509).

#### Social

For the social situation during *BSCT in general*, most patients reported feeling supported by the family, saying that everyone “supported” (#53, #25) and “complimented” (#53), that “family motivated stopping” (#106) and “nearly no one in our family smokes” (#14), that “my partner stimulated” (#509, #10), “offered incentives” (#14, #53), “accompanied” (#14), “gave feedback on better health conditions” (#53) and “does not smoke” (#509), and that “children supported” (#27), “children were positive about quitting” (#53) and “my son also quit” (#14). One patient said he (#25”) “lives alone” and “did not tell much about BSCT”; she reported that “everyone was sceptic of the quitting success.” One patient (#27) reported that “her partner did not support,” “questioned the Web sessions,” and broke “the agreement to smoke outside only.”

For friends and colleagues, the patients reported that “none of my friends smoke” (#10), “no one smokes inside” (#14), and that “colleagues also have positive experiences with cessation treatment” (#53). Furthermore, one patient emphasized that he “stimulates others to quit smoking” (#10).

### Consequences

Overall, *BSCT in general* had a positive *appeal*, while *emotions* (eg, “satisfaction”) varied. Again, there was clear distinction between the *F2F sessions* and the *Web sessions*. Similar to the emotional consequences, the behavioral consequences (*adherence*, *quitting*) also varied, ultimately resulting in diverse combinations of consequential appeal, emotions, and behavior.

#### Appeal

For six patients (#106, #53, #27, #75, #14, #25), *BSCT in general,* appealed to be “good.” The patients reported that BSCT was a “mix of talking and reading” (#14) and it “offered variety” (#75). The “shared information both F2F and Web was fine” (#106) and “Web only would not have been so easy” (#53). F2F sessions and Web sessions were “quite different” (#34); “sometime F2F was better—sometimes Web was better” (#14) and “Web was an extension of F2F” (#53). One patient (#27) emphasized the medical treatment saying “Champix was good.”

The *F2F sessions* mostly appealed to be “good.” The patients reported that the F2F sessions were “fine” (#509) or “finer than web” (#106) and that the F2F sessions were “most important” (#53) or “most important at treatment start” (#34). One patient (#12) emphasized “that only F2F touches your heart” and that he would go for F2F “100% in all facets.” However, one patient (#27) said that the F2F sessions were “whiny.” For the counselors, one patient (#27) described her counselor as “nice,” while another patient (#34) said that his counselor had a “stiff posture” and that she was “annoying,” “pedantic” and “cumbersome.”

For the *Web sessions*, the majority of patients reported a negative appeal, saying that the Web sessions “yielded nothing” (#509, #75, #14), were “a lot” (#509, #27), “cumbersome” (#106), “boring” (#34, #27), “tiring” (#27), “nonsense” (#12, #10), and “dead” (#10). However, one patient (#75) said that “Web was nice” while others—also referring to positive appeal—reported that the Web sessions could be done “comfortable at home” (#34) and that Web was “a serious matter” (#25), although she would not go for “Web only.”

#### Emotion

Emotional consequences varied—some patients were satisfied with *BSCT in general*, some not. Again, there was a distinction between *F2F sessions* and *Web sessions*, but not as clear as for the appeal.

While two patients (#34, #25) said that they were not satisfied with *BSCT in general*, three patients reported to be satisfied (#27, #10) or “thankful” (#106). Furthermore, referring to negative emotions about BSCT in general, patients reported “feeling abandoned, left alone” (#12), “tension and the need to relax physically” (#75), and “contradictions between quitting smoking and weight reduction” (#27). One patient said that the F2F sessions and Web sessions stimulated “the same moods” (#25). The mood during the *F2F sessions* was “good” (#53, #27), while *Web sessions* were experienced as “unpleasant” (#27) and “making me nervous” (#34). One patient reported to feel “guilty because I did not stick to appointments” (#27).

#### Behavior

During the interviews, three patients (#14, #53, #25) reported that they *adhered* to *BSCT in general,* doing both the F2F sessions and the Web sessions. One of them (#14) said he “could have stopped after four sessions” because he was “sure not to need it in the future.” However, he continued BSCT “to do the counselors and researchers a favor.” Five patients (#106, #34, #27, #10, #25) reported that they found the *Web sessions* “sloppy.” Furthermore, one patient (#27) mentioned that she “forgot about some of her sessions.”

Based on the auxiliary data (Table 2), medium adherence to *BSCT in general* (range 0-18, higher number indicate higher adherence) was 6.5 (IQR 5.50-11.75). Based on a 60% threshold for both the *F2F sessions* and the *Web sessions* [15], three patients (#14, #53, #106) were classified as adherent to BSCT in general. One patient (#75) was classified as adherent to the Web sessions but not to the F2F sessions, while another patient (#25)—one of the patients who reported to be adherent to BSCT in general during the interview—was classified as adherent to the F2F sessions but not to the Web sessions. Five patients (#509, #34, #27. #12, #10) were classified as nonadherent, because they neither adhered to the F2F sessions nor to the Web sessions.

Based on the interviews and the auxiliary data, four patients (#10, #14, #106, #53) reported successful *quitting*. One (#106) mentioned that “I had no problems because I had medication (Champix)” and “I threw away my last shags.” The other one (#53) mentioned that he told himself “Never again!” and “Enough!” (Basta!), and that “he saved money for the holidays with his family.” Two patients (#75, #509) reported that they reduced smoking during BSCT.

#### Combinations of Consequential Appeal, Emotions, and Behavior

The variety of consequential appeals, emotions, and behavior could be summarized in three types of combinations: “positive,” “negative,” and “mixed” consequences.

Three patients (#14, #53, #106) experienced “positive” consequences. BSCT appealed to be good and they felt “satisfied”/“thankful,” adhered to the treatment and quit smoking.

On the contrary, another three patients (#12, #34, #509) experienced “negative” consequences: The Web sessions appealed negative (“nonsense,” “boring,” and “yielded nothing”) and BSCT in general resulted in negative emotions (abandoned/not satisfied). Ultimately, they did not adhere to the treatment and did not quit smoking.

Mixed consequences: Three (#25, #27, #75) of the four remaining patients did not quit smoking, while one (#10) did. Interestingly, BSCT in general appealed “good” to the nonquitters (#25, #27, #75) while—for the quitter (#10) at least, the Web sessions appealed to be “nonsense.” Although two of the nonquitters (#25, #75) reported negative emotions (“tension”/“not satisfied”), these two patients at least partly adhered to BSCT (#25 adherent to F2F sessions; #75 adherent to Web sessions). In turn, the third nonquitter (#27) reported positive emotions (“satisfied”) but did not adhere at all.

To the remaining quitter (#10), although the Web sessions appealed to be “nonsense” and he did not adhere to BSCT in general, he reported positive emotions (“satisfied”) and ultimately quit smoking.

## Discussion

### Principal Findings

This study aimed to provide insight in the UX of a blended treatment. In the light of this study, the expectation that the strength of one mode of delivery can compensate for the weaknesses of the other in blended treatment, can be partially supported because the F2F sessions compensated for the weaknesses of the Web sessions so that BSCT in general was mostly experienced positively.

Our study described the UX of a BSCT using Hassenzahl’s key elements of UX from a user’s perspective [23,24]. Overall, BSCT in general appeared to be a mostly positively experienced service. Patients had a positive-pragmatic standard and neutral-open expectation toward BSCT in general at treatment start, and the pragmatic attributes of the F2F session were mostly perceived as positive while the pragmatic attributes of the Web sessions were perceived as both positive and negative. For the hedonic attributes, there seems to be a difference between the F2F and Web sessions. Specifically, the hedonic attributes of the Web sessions were experienced mostly negative while the hedonic attributes of the F2F sessions were mostly positive. For the usage situation, the physical and social context was experienced positively while the task and technical context was experienced negatively. Nevertheless, the consequential appeal of BSCT in general was positive. However, the consequential emotions and behavior varied, ultimately resulting in diverse combinations of consequential appeal, emotions, and behavior (positive, negative, and mixed).

Although patients’ pretreatment expectations toward BSCT were neutral and the Web sessions appealed negative, overall BSCT in general appeared to be positively experienced afterwards. This is in line with an evaluation study (n=7) by Kooistra et al [14] of a blended cognitive behavioral treatment for major depression. However, our study provides a more differentiated insight in why the Web sessions were appraised negatively. Applying Hassenzahl’s distinction between pragmatic and hedonic attributes [23], our findings suggest that while patients experienced the pragmatic attributes (usability, utility) of the Web sessions in general as more positive, the negative hedonic attributes (stimulation, identification, and evocation) of the Web sessions led to a combination of negative consequences, such as negative appeal, negative emotions, and low adherence.

Interestingly, although the hedonistic gap made the Web sessions appeal negatively, the overall BSCT was experienced positively. This could support the assumption that in blended treatment the strength of one mode (ie, F2F) may compensate for the weaknesses of the other (ie, Web) [4,5]. This is further supported by our findings about relatedness and identification, which are in line with a qualitative study (n=14) by Wilhelmsen et al (2013) [22] on the internet-based cognitive behavioral therapy for depression supported by short F2F consultations. Three of our patients that adhered to BSCT and ultimately quit smoking showed rather low identification with the Web sessions but had positive appeal and emotions toward BSCT in general, especially toward the F2F sessions. This positive overall appraisal may have cancelled out the negative appeal of the Web sessions.

We mainly found that the F2F sessions compensated for the weaknesses of the Web sessions. Yet three patients reported that the Web sessions influenced their personal contact with the counselor positively. Although the Web sessions mostly had a low identification and a negative appeal, the Web sessions supported the F2F sessions because these patients felt more related to the counselor. However, even though the Web sessions may have supported the F2F sessions, it should be noted that none of the patients indicated that the Web sessions compensated for the F2F sessions. It remains undecided if this is because there was no need for compensation as the F2F sessions were overall positive, or that the Web sessions were not able to compensate. It should also be taken into account that the routine care in the hospital was not Web based, the patients were of older age, and did not have an affinity for the internet (although they reported to have sufficient internet skills), and the patients’ preferences for modes of delivery were not taken into account as they were not free to choose for BSCT because they were included in a randomized controlled trial. These factors may additionally explain the low positive impact of the Web sessions.

The emotional and behavioral consequences varied, ultimately resulting in three types of combinations of appeal, emotions (eg, satisfaction), and behavior (adherence, quitting): “positive,” “negative,” and “mixed.” These types can be used to work on UX profiles that can support further development of blended care and improve the matching between the treatment and patient [3].

### Implication for Future Research and Clinical Practice

Further work needs to be done to investigate how the integration of F2F and Web treatments can be carried out to ultimately increase the effectiveness and efficiency of a blended treatment. This study provides a hint to explore this question by emphasizing the relevance of hedonic attributes in the UX. Even if the UX was predominantly positive because the hedonistic gap in the area of the Web sessions was compensated relatively easily by the F2F sessions, this does not mean that BSCT cannot be further improved to increase adherence and long-term abstinence. Hedonism could be a starting point for this. Further research on the following questions could be useful:

Could the hedonistic gap in the Web sessions be not only due to the mode of delivery, but also the concrete content of the Web sessions? Perhaps, it was precisely the interventions that the patients experienced as nonhedonistic, which were the part of the Web sessions. This was neither explicitly considered in the treatment design nor asked for in detail in the interviews. However, this might have been the case because more standard exercises and messages could be offered on the Web more easily. A stronger involvement of patients in the early design stages of the Web sessions may help to prevent the hedonistic gap.

May hedonism play a less prominent role in the health care context than in the other domains? Patients tend to approach a health problem with a pragmatic-neutral expectation, such as “What’s important is that it works. As long as it helps, I can also accept that it is unpleasant.” Consequently, hedonistic aspects, such as fun, enjoyment, pleasure, and aesthetics may not be expected in the first place, and therefore, may not be missed. Moreover, this may be compensated relatively easily by positive experiences with the counselors. However, if hedonism was less important in health care, it would contradict our conclusion that it should receive more attention.

Could both scientific research and clinical practice use insights from persuasive systems design [36,37], “nudging” [38] and “funology” [23,24] to address the hedonic gap, which may negatively influence smoking cessation patients who are usually a highly motivated target group [39]? Persuasive design features, such as primary task support (eg, tailoring, personalization), dialogue support (eg, rewards, liking), credibility support (eg, real-world feel), social support (eg, normative influence, competition), and hedonic aspects (eg, fun, enjoyment, pleasure, and aesthetics) may play a role in sustaining patients’ motivation to adhere to the treatment and quit smoking.

How do the apparent character and the consequential appeal and emotions relate to the quitting behavior? On the one hand, apparently a negative appeal (ie, missing hedonic attributes) may lead to consequential combination of negative appeal, emotions, and behavior (ie, neither adhere nor quit). On the other hand, it is also possible to distinguish between diverse episodic UXs ultimately leading to a cumulative UX [20], for example, a motivated patient may start with a positive UX but after failing to quit or relapsing, the patient’s standards and expectations may change during the treatment, which can then lead to a negative appeal and ultimately to a cumulative negative UX. The cumulative UX would then not be the result of a linear process as in the model of Hassenzahl (Figure 1). Rather, in a circular process, consequences (ie, quitting), apparent character, expectations, and standards would influence each other.

### Strengths and Limitations

The data and model used in this study provided a rich insight into the UX of a blended treatment for smoking cessation in an ambulant clinical setting. Though this study yielded valuable knowledge for the understanding and improvement of BSCT and the matching of patients and treatment, limitations should be noted when interpreting the findings. First, the sample of patients used in this study was a purposive sample that was intended to represent the heterogeneity of the patients of an outpatient cessation clinic. Hence, it is uncertain, if the rather small sample (n=10) is representative of the population referring to characteristics, such as sex, age, internet skills, or educational level, and if the thematic saturation was reached with this sample size. It should also be considered that patients did not choose BSCT on their own but were randomly assigned to it because they participated in a randomized controlled trial. However, the high degree of consensus in the findings may indicate generalization of our main conclusions. Second, the interviews were conducted retrospectively. Conducting additional interviews at treatment start and during treatment could have offered a more valid insight in the process of patients’ UX construction (eg, for the standards, expectations, and apparent character). Third, the software that was used for the Web sessions was developed around 2005, which may have led to technical incommodities (eg, Web software is “Flash”-based and nonresponsive; not mobile device–compatible), which may have negatively impacted the UX. This assumption is based on the fact that patients often stated that they would have liked to do the Web sessions on their mobile device. We assume that a newer mobile device–compatible software with similarly good pragmatic attributes as the previous Flash-software could have also improved the hedonistic attributes and thus have led to more positive consequences. Fourth, the interviews were conducted with the first patients that followed the new blended version of the smoking cessation treatment. At that time, the treatment still had some teething problems, such as being new for the originally F2F counselors. We did not integrate the counselors’ views on the uptake of BSCT, and therefore, we cannot compensate for bias through inadequate treatment fidelity. Fifth, as long-term abstinence is the goal of a smoking cessation treatment, prolonged follow-up analysis of patients’ UX could reveal a different picture. For example, some patients may continue using the Web-based modality and benefit from this at a later stage, resulting in a UX that would be more in favor of the Web-based treatment. Conversely, relapse to smoking at a later stage may lead to a negative adjustment of the UX of the blended treatment. Sixth, we could not elaborate further on which specific parts of the Web sessions were experienced positively or negatively as we did not ask for these in detail in the interviews. Seventh, the study interventions are selected and combined by the researchers and treatment developers without considering the individual patients who ultimately followed the treatment. This resulted in a rather inflexible approach of blending (five Web-based sessions and five F2F sessions in a fixed sequence and with equivalent content) to allow for comparisons with the F2F treatment as usual in the randomized controlled trial (LiveSmokefree study) [8]. This inflexible approach is due to the research design and may limit the potential of blending. In daily practice, the blending of Web-based and F2F interventions may lead to a flexible exchangeability of all intervention components, which would foster a treatment that is highly tailored to the patient’s needs and abilities and could lead to a different UX.

### Conclusions

This study provides insight into the key elements of the UX of a blended treatment for smoking cessation and supports the expectation that in a blended treatment, one mode of delivery may compensate for the weaknesses of the other. However, in this certain setting, this could be mainly achieved in only one way: F2F sessions compensated for the weaknesses of the Web sessions. As a practical conclusion, this may mean that the Web sessions supported by the strength of the F2F sessions, offer an interesting approach for further improving the blended treatment in this specific context. Our theoretical findings reflect the relevance of the aspects of hedonism, such as fun, joy, or happiness in UX [23], which were not mentioned in relation to the Web sessions and only scarcely mentioned in relation to the F2F sessions. Future research should further investigate the role of hedonistic aspects in the blended treatment and if increased enjoyment of the blended treatment could increase the treatment adherence and ultimately its effectiveness.

## Appendix

Multimedia Appendix 1 Screenshots of the Web sessions of Blended Smoking Cessation Treatment.

Multimedia Appendix 2 Blended Smoking Cessation Treatment user experience Interview Guide (Dutch).

Multimedia Appendix 3 Codes, code description, and intercoder agreement per semantic domain.

Multimedia Appendix 4

## Abbreviations

BSCT: Blended Smoking Cessation Treatment
eHealth: electronic health
F2F: face-to-face
SRP: Outpatient Smoking Cessation Clinic (Dutch: Stoppen met Roken Poli)
UX: user experience
