# Supplementary material for: Patients’ User Experience of a Blended Face-to-Face and Web-Based Smoking Cessation Treatment: Qualitative Study
Source: JMIR Form Res. 2020 Jun 3;4(6):e14550. doi: 10.2196/14550 (PMC7301265; doi:10.2196/14550)

# Multimedia Appendix 1. Screenshots of the web-sessions of BSCT

In the following, screenshots of the web-sessions of BSCT are provided in order to give an impression of its look and feel. Note: The language of the web content is Dutch. Therefor a comprehensive English description is added.

## Landing page

The landing page at www.rokendebaas.nl offers the possibilities to register and log in, to follow a “demo-treatment” and to find information about smoking and smoking cessation.

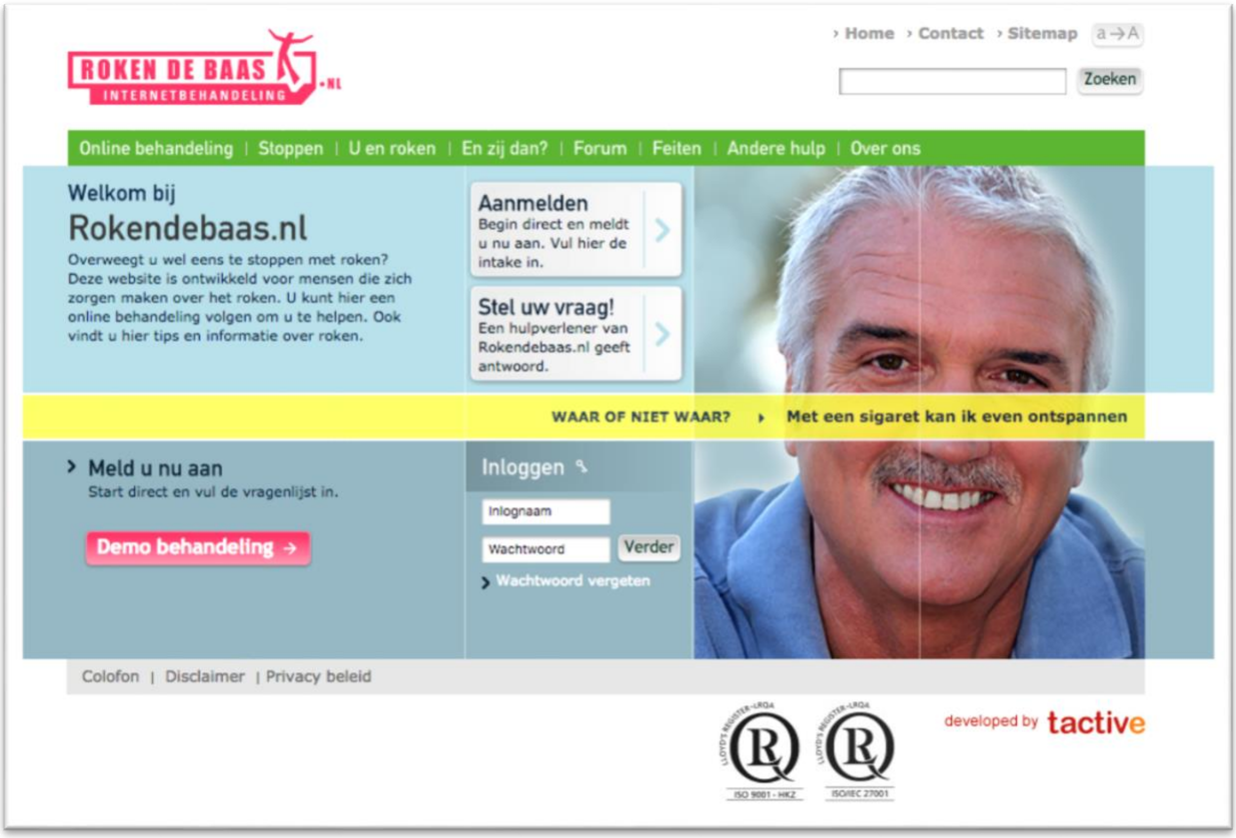

## Starting page of the treatment

Once logged in, patients choose to proceed with going to their record (“Dossier”), to the smoking self-recording (“Rookschrift”), or the to the forum to exchange with peers. In addition, if new messages (from example from the counselor) are available is also displayed here (“Berichten”).

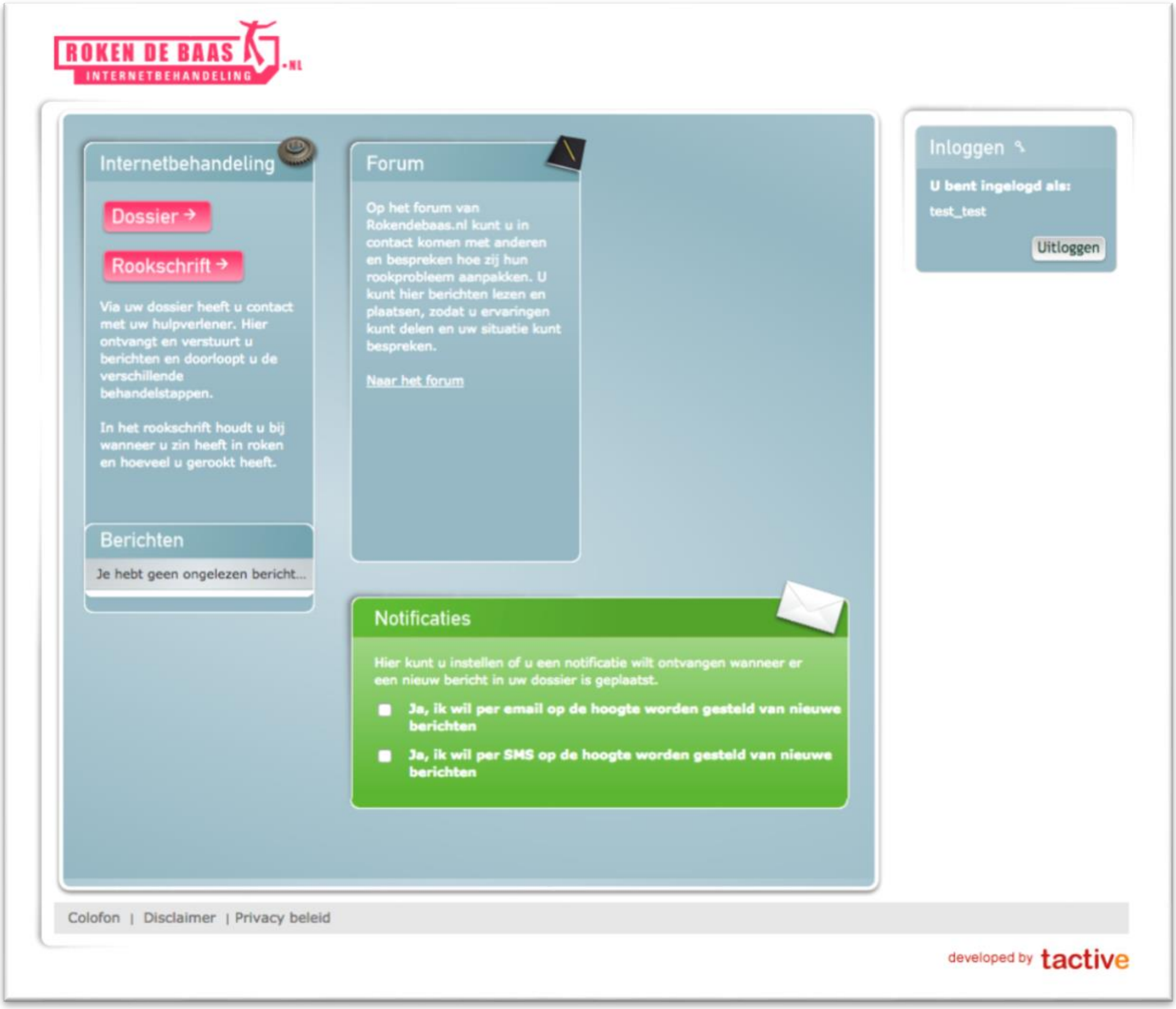

## Inbox

This is the inbox of the integrated messaging system for patients and counselors. In addition, patients can go directly to their smoking self-recording (Rookschrift") and to the interventions which are already unblocked by the counselors. In this case, the patient could go directly to "Goal setting ("Doel stellen - … ") or to "Measuring and Knowing ("Meten en weten".

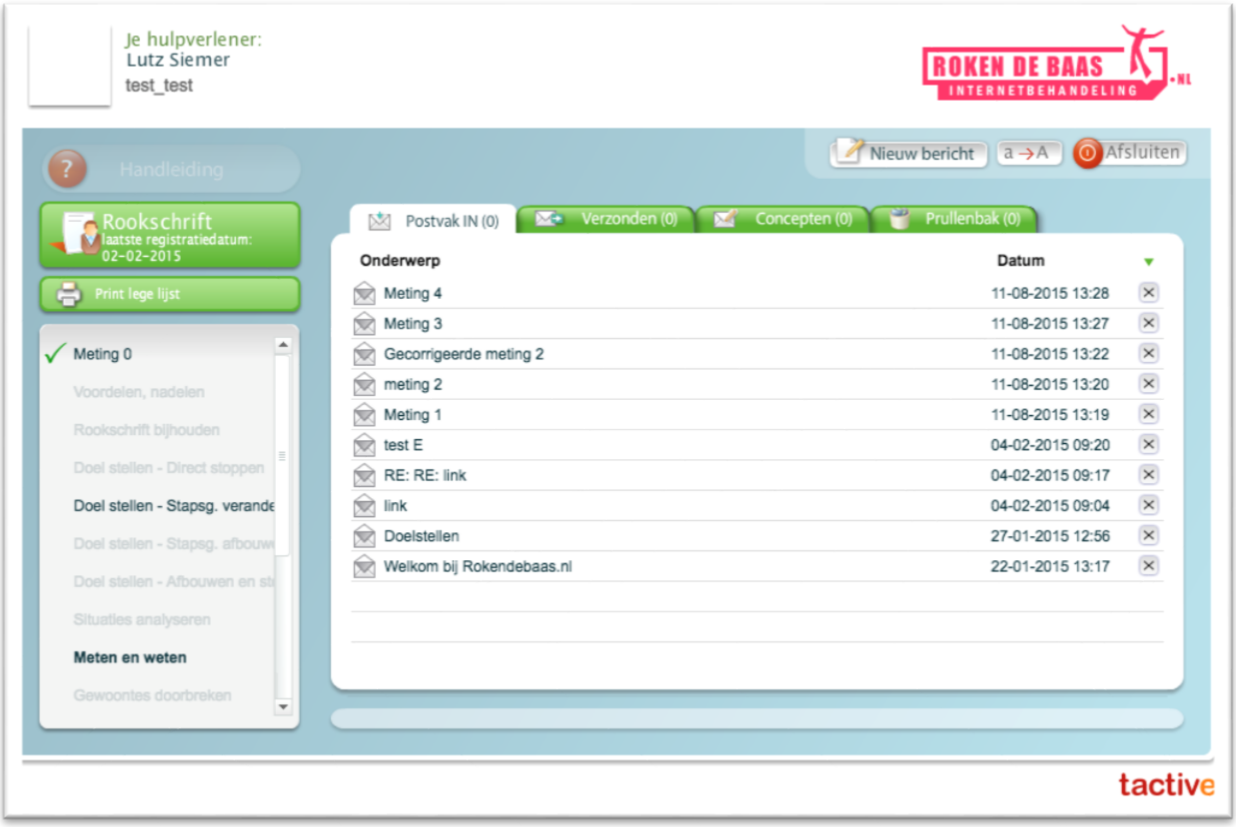

## Reading messages

The inbox after opening a message. Patients have the option to reply, print or close the message.

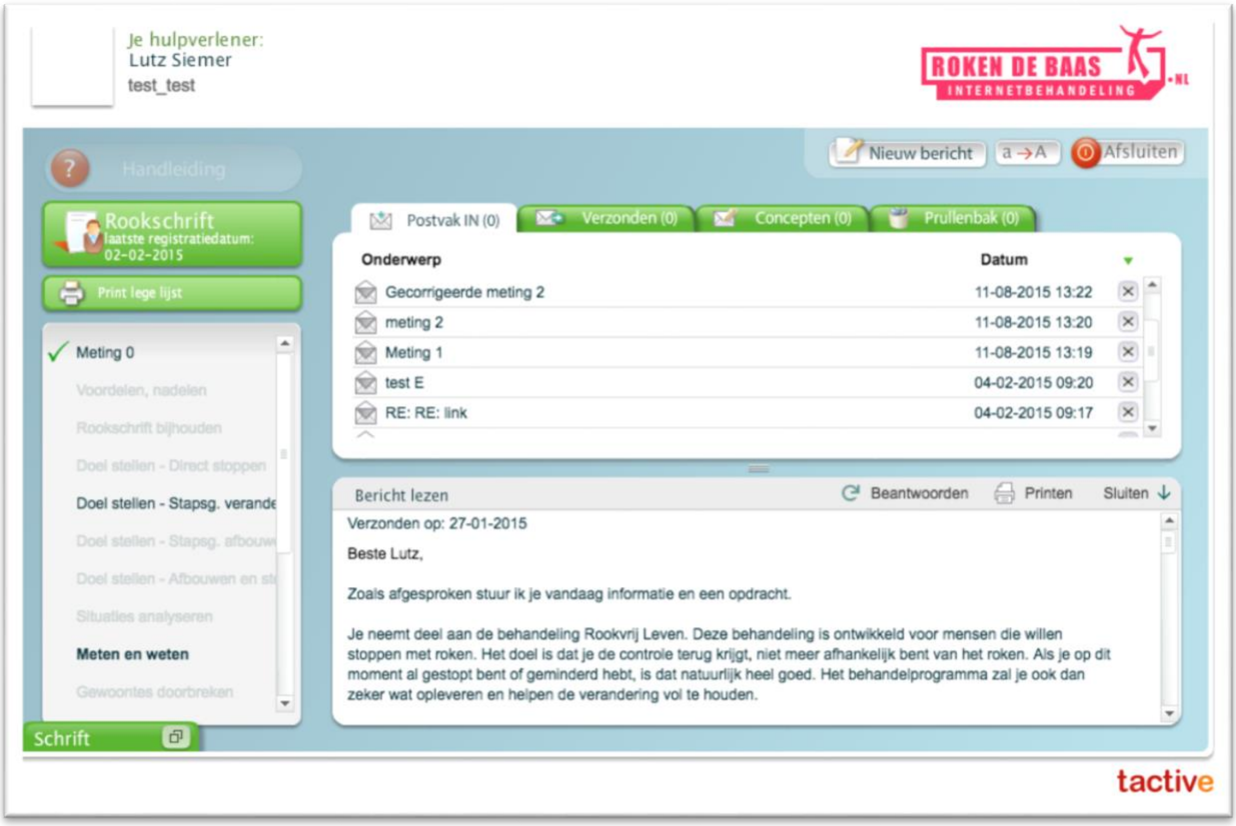

# Goal setting

This is the first page of the goal setting intervention where the patient is ask to think about what he/she wants to achieve precisely. In addition, four tips for goal setting are provided.

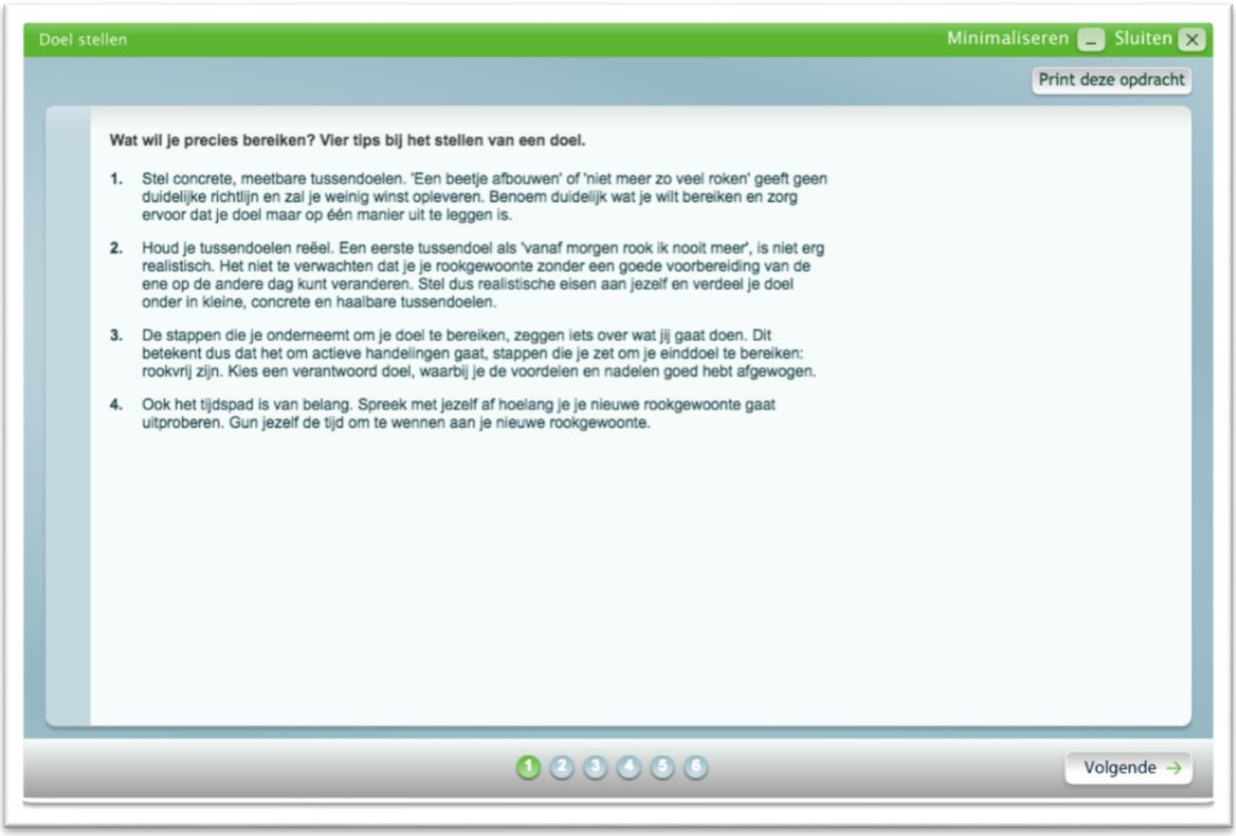

## Why do you want to stop smoking (“Waarom wil je stoppen met roken”)?

The second page of the goal setting intervention where the patient is asked to fill in as many reasons as possible and to do this as precisely as possible.

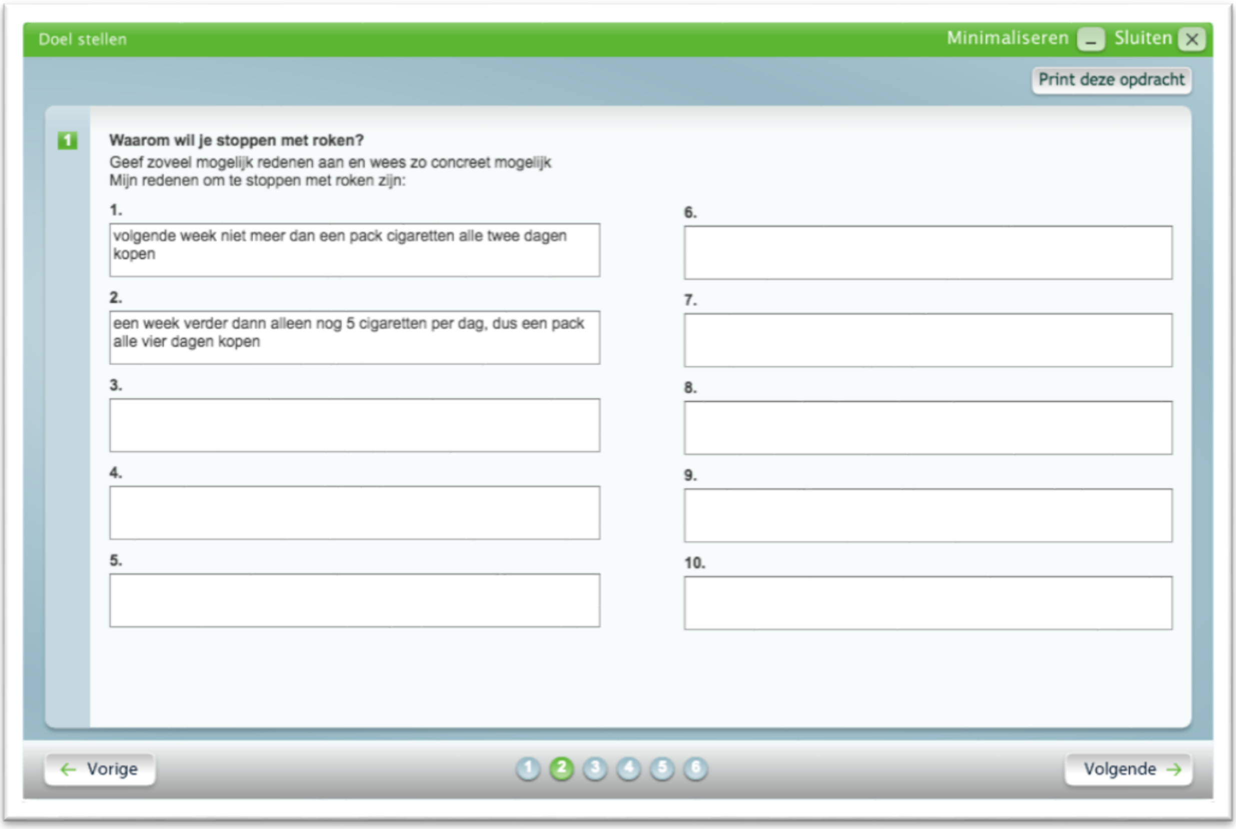

## Final goal ("Einddoel")

Third page of the Goal setting intervention, where the patient is asked if he/she will fix a stop date (Einddoel") and which smokefree activities he/she will do when ("mijn volgenden stap naar mijn einddoel").

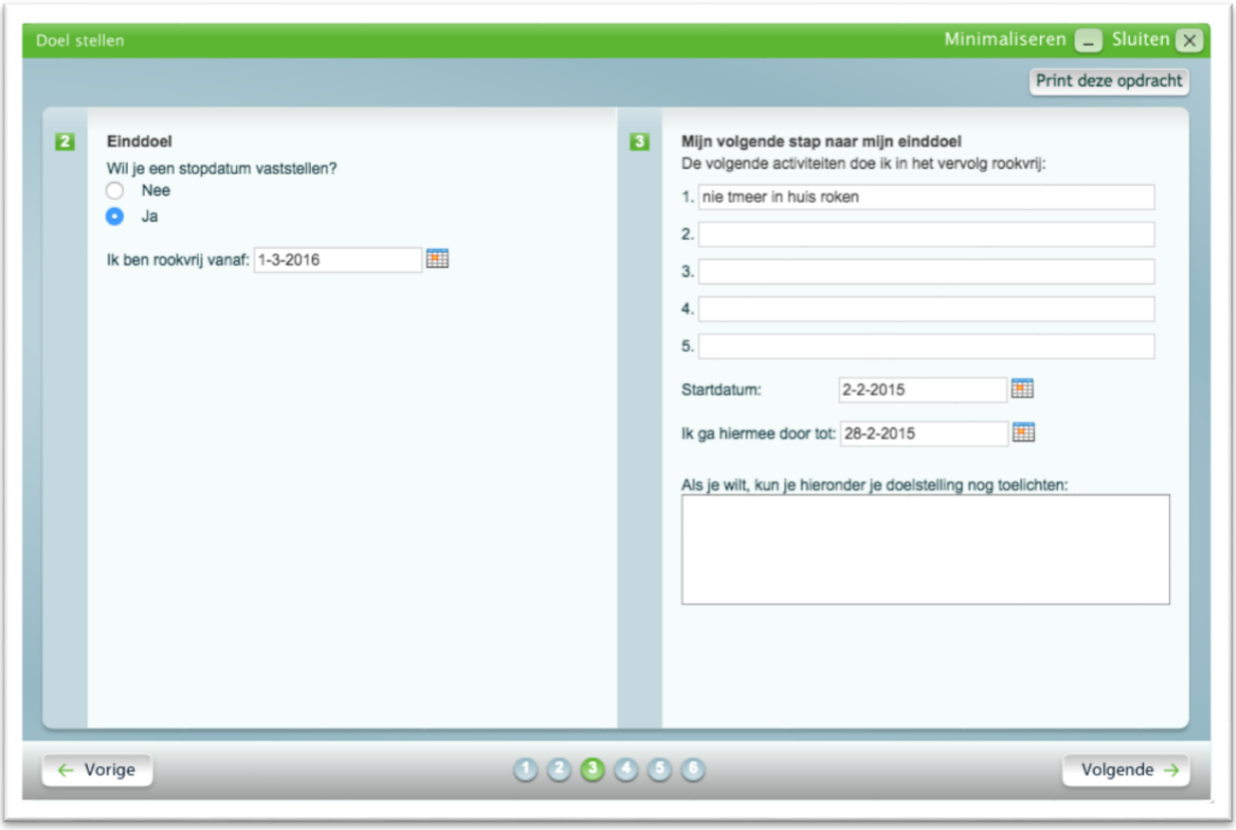

## More goals

Fourth page of the goal setting interventions where the patient may add more goals and activities.

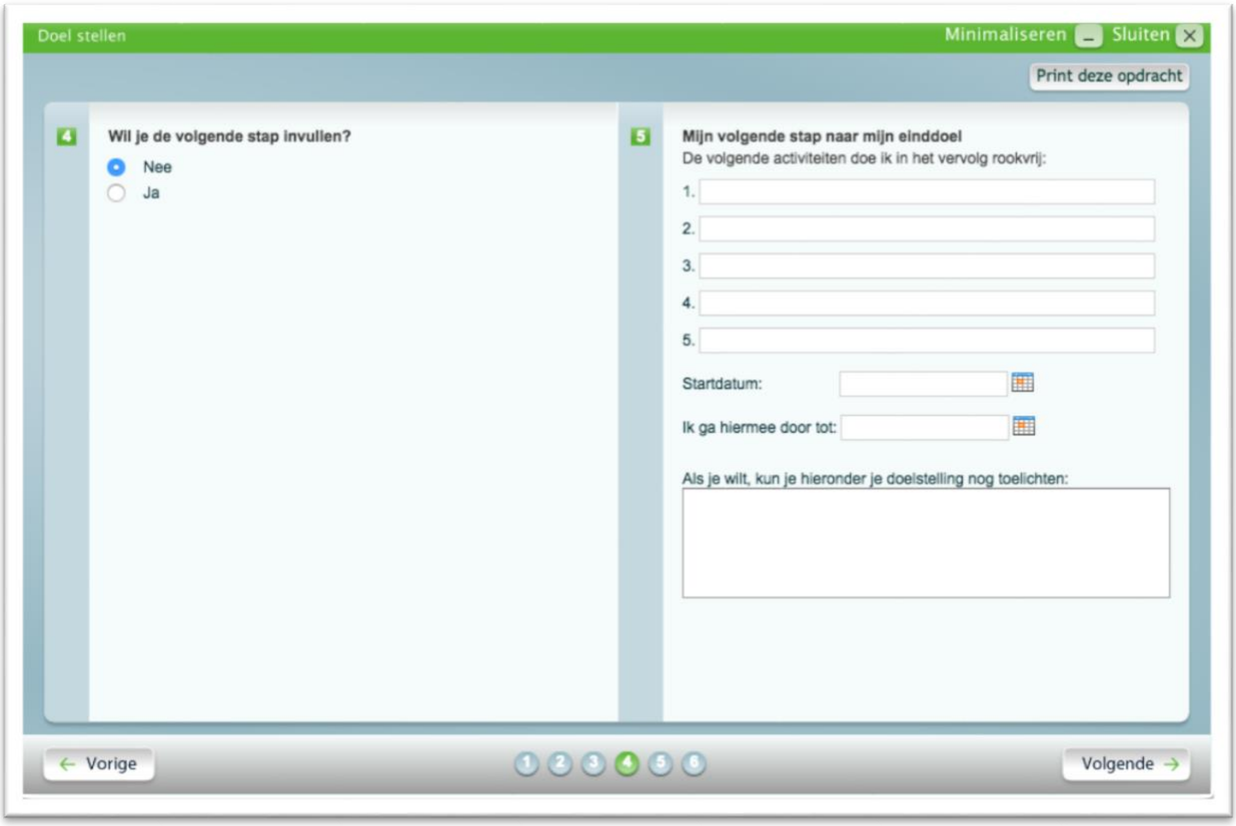

# Smoking self-recording

Overview of the smoking self-recording ("Overzicht registratie") where the patient's registry of numbers of cigarettes and the moment descriptions are displayed.

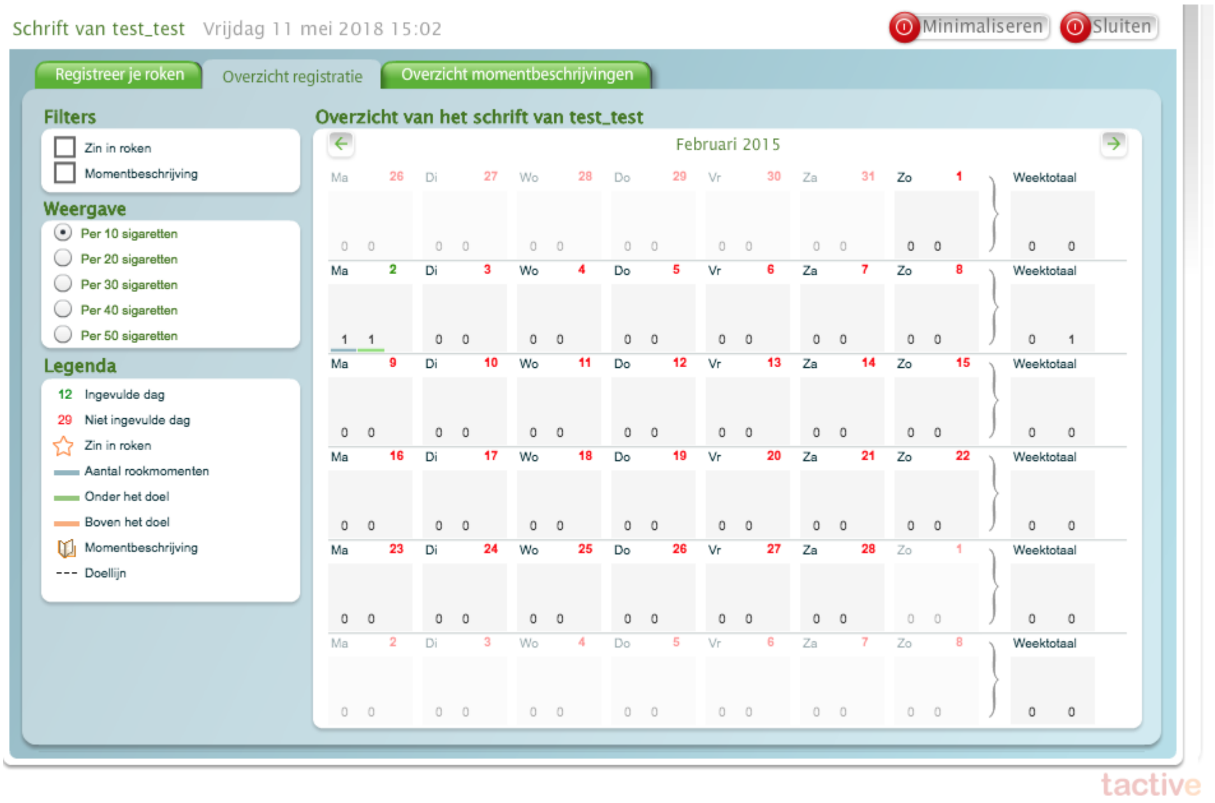

# Moment descriptions

Here the patient can record the following characteristics for a smoking situation:

- Time of day
- Level of craving
- Cigarette brand
- Number of cigarettes smoked
- Level of smoking pleasure
- Situation (where, alone/not alone, busy with what, event)
- Thoughts
- Emotions
- Behavior

Furthermore, patients can print moment descriptions (either all or per day).

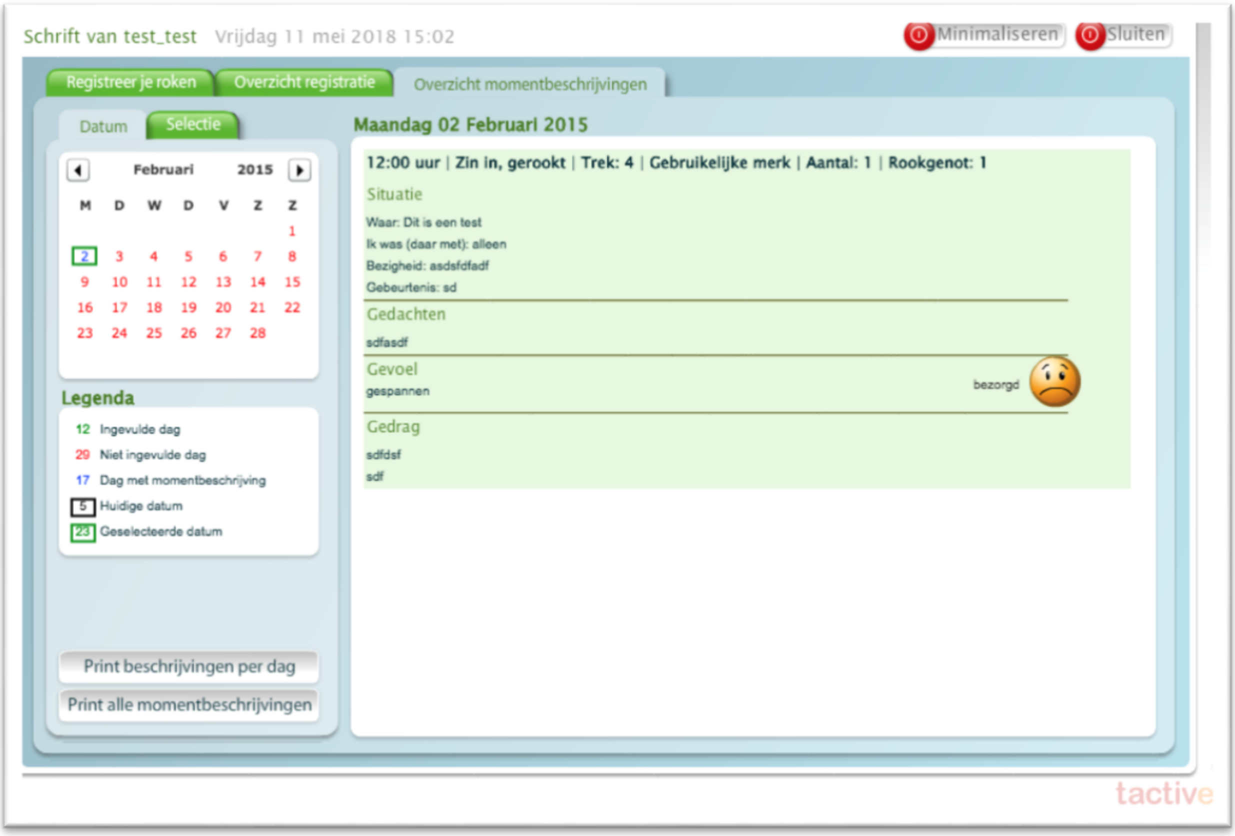

Supplement: Multimedia Appendix 1 [file formative_v4i6e14550_app1.pdf]
