# Supplementary material for: Patients’ User Experience of a Blended Face-to-Face and Web-Based Smoking Cessation Treatment: Qualitative Study
Source: JMIR Form Res. 2020 Jun 3;4(6):e14550. doi: 10.2196/14550 (PMC7301265; doi:10.2196/14550)
Supplement: Multimedia Appendix 2 [file formative_v4i6e14550_app2.pdf]

THEMALIJST EN VRAGEN UX BSCT

Voorbereiding en introductie 2
Voorbereiding 2
Introductie 3

Vragenlijst 4
Algemene ervaringen met de combi behandeling 4
Verwachtingen en ervaringen vooraf 5
Gebruiker 6
Motivatie bij het volgen van de combi behandeling 6
Stemming bij het volgen van de combi behandeling 7
Ressources tijdens het volgen van de combi behandeling 7
Context 8
Sociale context tijdens het volgen van de combi behandeling 8
Fysieke context tijdens het volgen van de combi behandeling 8
Taak context tijdens het volgen van de combi behandeling 9
Technische en informatieve context tijdens het volgen van de combi behandeling 9
Evaluatie 10
Gebruiksvriendelijkheid van de combi behandeling 10
Tevredenheid met de combi behandeling 12
Verdeling face-to-face en online gedeeltes 12
Voordelen/nadelen 13
Afsluiting 14
Eigen toevoeging van de patiënt 14
Omschrijving combi behandeling 14

Afsluiting 15

Rapport van de interviewer 16

Aanvullende data over patiënt (uit b.v. eerder ingevulde vragenlijsten of DSV) .... Fehler! Textmarke nicht definiert.

Termen die die counselors gebruiken

- “Combi” behandeling
- “Online”
- “afspraken op de poli”

VOORBEREIDING EN INTRODUCTIE

VOORBEREIDING

- Stel voor om aan een tafel plaats te nemen, bij voorkeur in een kamer waar geen anderen aanwezig zijn.
- Ga zo zitten dat de respondent moeilijk kan meelezen met wat er op papier staat of wat je opschrijft (bijvoorbeeld aantekeningen).
- Geef aan dat je het interview graag wilt opnemen en dat de opname alleen wordt gebruikt om een gespreksverslag te maken en dat de opname na afloop van het onderzoek wordt vernietigd.
- Vraag aan de respondent of hij/zij het goed vindt dat het gesprek wordt opgenomen.
- Pak de nodige materialen en zet de opnameapparatuur klaar
- Geef aan dat je, voordat je van start gaat met het interview, een testje wil doen met het opnameapparaat.
- Schakel het apparaat in spreek in: “test”, de datum van vandaag en het tijdstip”
- Vraag aan de respondent of hij de datum en het tijdstip wil herhalen.
- Spoel de opname terug en verzeker jezelf ervan de geluidsopname goed is. Pas zo nodig het geluidsniveau en/of de opstelling aan en herhaal de test totdat de geluidsopname goed is.
- Kondig aan dat je klaar bent om met het interview te beginnen.

INTRODUCTIE

“(Nogmaals) hartelijk dank dat u mee wilt werken aan dit interview. Wij verwachten dat dit interview tussen 60 en 90 minuten gaat duren. Door het afnemen van interviews proberen we inzicht te krijgen in de ervaringen van deelnemers aan de gecombineerde online en face-to-face behandeling. In de rest van dit interview zullen wij deze gecombineerde behandeling “de combi behandeling” noemen. De uitkomsten geven ons meer inzicht in uw ervaring met de combi-behandeling voor stoppen met roken en helpt ons waar nodig deze behandeling te verbeteren.

“Het interview heeft geen invloed op uw werkzaamheden op de afdeling Longgeneeskunde. Uw collega's en leidinggevenden, bijvoorbeeld het teamhoofd, krijgen geen informatie over wat u hier vertelt”.

“Uw persoonsgegevens worden strikt vertrouwelijk behandeld en de gegevens die u tijdens het interview verstrekt worden anoniem verwerkt en zijn op geen enkele manier tot u te herleiden.”

“Zoals u weet zijn we geïnteresseerd in de ervaringen van de mensen die met de combi behandeling voor het stoppen met roken gewerkt hebben tijdens de RookvrijLeven-studie en zijn we benieuwd hoe zij dit, ieder op hun eigen manier, hebben ervaren. We willen u daarom vragen om uw ervaringen met de combi behandeling te vertellen; we horen graag alle ervaringen en gebeurtenissen die daarbij belangrijk waren voor u. “

“Dus uw mening over en beleving van de behandeling staat centraal bij dit interview.”

“Neemt u alstublieft alle tijd die u nodig heeft. Wij zullen vooral luisteren en proberen om u niet te onderbreken. Wij zullen af en toe aantekeningen maken.”

“Heeft u nog vragen vooraf?“

VRAGENLIJST

ALGEMENE ERVARINGEN MET DE COMBI BEHANDELING

Themalijst:

Ervaringen algemeen	❑
Dingen die goed/slecht gingen	❑
Ervaringen met de patiënt f2f	❑
Ervaringen met de patiënt online	❑
Verwachtingen algemeen	❑
Verwachtingen online	❑
Verwachtingen face-to-face	❑
Verwachtingen patiënt	❑
Eerdere ervaringen combi	❑
Eerdere ervaringen online	❑
Eerdere ervaringen computer & internet	❑
Zelfbeschrijving computergebruik	❑
Ervaring blended communiceren	❑
Invloed ervaringen	❑

“KUNT U ONS, OM TE BEGINNEN, VERTELLEN WAT UW ERVARINGEN ZIJN MET DE COMBI BEHANDELING? ALLE GEBEURTENISSEN EN ERVARINGEN DIE BELANGRIJK VOOR U WAREN HOREN WE GRAAG.

Doorvragen kan altijd door…

“U noemt …. . Kunt u er meer over vertellen?”

“U noemt … . Kunt een voorbeeld geven?”

"Zijn er naast de dingen die goed gingen ook dingen die minder goed gingen?"

"Zijn er naast de dingen die minder goed gingen ook dingen die juist wel goed gingen?"

Vragen over verandering tijdens de behandeling (vooraf, begin, tijdens, aan het eind, nu terugkijkend), b.v.

“U noemt ….Was dat ook aan het begin al zo?”

“Is er gedurende de studie iets veranderd in uw opvatting over… ?”

Volgende vraag alleen stellen als de respondent veranderingen noemt.

„U noemt … Wat maakt dat er een omslag is geweest in uw ervaring? Op welk punt van de studie was dit ongeveer?“

“Kunt u zich de reden hiervoor nog herinneren?”

Doorvragen naar counselor tegenover online.

"Wat zijn uw ervaringen met het contact met de patiënt op de poli?"

"Wat zijn uw ervaringen met het contact met de patiënt online?"

[Doorvragen.. zijn hier tegenstrijdigheden in?]

VERWACHTINGEN EN ERVARINGEN VOORAF

> “Wij willen graag iets dieper ingaan op uw verwachtingen van de combi behandeling en ervaringen voorafgaand aan het RookvrijLeven-traject”
>
> “Welke verwachtingen had u van de combi behandeling toen het duidelijk was dat u met deze vorm van behandelen ging werken?“

“Welke verwachtingen had u van het online gedeelte van de behandeling?”

“Welke verwachtingen had u over de afspraken op de poli?”

“Welke verwachtingen had u over de patiënt?“

„Welke eerdere ervaringen had u met een online behandeling/behandelen?”

„Welke eerdere ervaringen had u in het algemeen met computer en internet?”

“Hoe zou u zichzelf beschrijven als het gaat om computergebruik in het algemeen?”

„U kent nu de combi behandeling. Had u voorafgaand aan deze behandeling reeds ervaring met online communiceren in combinatie met face-to-face afspraken? Denk hier bijvoorbeeld aan afspraken via e-mail/vergaderen, whatsapp of iets dergelijks. Kunt u daar iets over vertellen?”

Evtl. doorvraag..

“Denkt u dat dit invloed heeft gehad op de combi behandeling?”

GEBRUIKER

Themalijst:

Reden stoppen/doorgaan met behandeling	❑
Combi motiveert stoppen met roken	❑
Helpende onderdelen	❑
Motivatie door counselor	❑
Patiënt leest informatie	❑
Stemming	❑
Verandering stemming	❑
Behandeling passend bij counselor	❑
Eigenschappen/vaardigheden voor het werken met de combi	❑

"Wij willen het nu graag over u als behandelaar hebben. We vragen bijvoorbeeld naar uw motivatie en/of u de behandeling voor u passend vind."

MOTIVATIE BIJ HET VOLGEN VAN DE COMBI BEHANDELING

"Wat maakt het dat u met de ene patiënt bent gestopt met de behandeling en met een andere patiënt juist doorgaat?"

[De volgende vraag alleen stellen als de respondent geen toelichting geeft op de reden voor stoppen/doorgaan]

"Kunt u toelichten waarom ……………….[reden(en) noemen] de reden was?"

De volgende vraag aan alle respondenten stellen.

"Waren er naast …[reden(en) noemen] voor u <u>nog andere redenen</u> om te stoppen/ door te gaan met behandelen?"

"Als u kijkt naar deze combi behandeling, in hoeverre <u>motiveerde</u> deze opzet u hiermee te werken?"

"Wat is voor u het meest motiverende onderdeel van de <u>combi</u> behandeling?"

"Is dat meer iets wat in het <u>face-to-face gedeelte of in het online</u> gedeelte gebeurd?"

"Welke <u>onderdelen</u> helpen u het <u>meest</u> bij het behandelen?"

"Welke <u>onderdelen</u> helpen u niet of <u>minder</u> bij het behandelen?"

"In hoeverre motiveren de reacties van de patiënten u? En maakte het voor u verschil of het een online contact of het een afspraak op de poli betreft?"

Evtl. doorvragen…

"Hoe bewust bent u van het feit dat de patiënt de online informatie gaat lezen en er daadwerkelijk wat mee doet? In hoeverre beïnvloed dat uw werkwijze?"

STEMMING BIJ HET VOLGEN VAN DE COMBI BEHANDELING

"Het werken met een nieuwe behandelvorm gaat meestal gepaard met de nodige stemmingen en emoties.

U kunt hierbij denken aan bij voorbeeld vreugde of verdriet, woede, angst, verbazing of walging. "

"Welke emoties heeft u met name ervaren?

"Zijn er veranderingen in uw gemoedstoestand geweest tijdens dit gehele traject?"

Als deelnemer iets noemt doorvragen …

"Wanneer was dat? Had dit meer te maken met het leren werken met deze behandel vorm of meer met een specifiek onderdeel van de behandeling?"

Als deelnemer alleen dingen noemt over het leren werken met de behandeling…

"Zijn er ook bepaalde onderdelen van de combi behandeling die meer effect hebben op je gemoedstoestand?"

Als deelnemer alleen dingen noemt over de onderdelen van de behandeling…

"Was er bij het leren werken met deze combi behandeling effect hebben op je gemoedstoestand?"

RESSOURCES TIJDENS HET VOLGEN VAN DE COMBI BEHANDELING

"Vindt u deze manier van behandelen passend bij u?"

„Waren er specifieke onderdelen die u vooral passend vind bij uzelf?"

„Om de combi behandeling goed aan te kunnen bieden is het belangrijk dat iemand o.a. voldoende computervaardig is. Herkent u dit? Wat zijn volgens u nog meer eigenschappen/vaardigheden die belangrijk zijn voor een counselor om een combi behandeling aan te kunnen bieden?"

De volgende vraag alleen stellen als de respondent geen toelichting geeft op de vaardigheden

"Kunt u toelichten waarom ……………….[vaardigheid noemen] nodig is?"

Eventueel doorvragen naar fysieke/mentale ressources

CONTEXT

Themalijst:

Mensen rondom heen	❑
Verschillende plekken	❑
Vrijheid vs. structuur	❑
Voorlichting	❑
Techniek	❑

"Wij willen ook graag weten wat er rondom de behandeling heen gebeurt. Wat zeggen collega's, leidinggevenden en andere mensen in uw omgeving over blended behandelen? Wat vond je van de behandelplekken – hier op de poli of online? We zullen met de mensen in uw omgeving beginnen."

SOCIALE CONTEXT TIJDENS HET VOLGEN VAN DE COMBI BEHANDELING

"In hoeverre werd uw ervaring met de combi behandeling beïnvloed door mensen in uw omgeving? Denk hierbij aan leidinggevenden, collega's, familie en anderen … wat zij dachten, zeiden of deden."

Wat zeiden….

- ***Collega's***
- ***Leidinggevenden***
- ***Andere medewerkers van het MST bijv. secretaresses***
- ***Familie en vrienden***
- ***Onderzoekers van de RookvrijLeven Studie***

"In hoeverre werd u gemotiveerd (of juist niet) door mensen in uw omgeving?"

FYSIEKE CONTEXT TIJDENS HET AANBIEDEN VAN DE COMBI BEHANDELING

"Op welke plekken heeft u de online onderdelen van de behandeling aangeboden? Waarom op deze plek?

"Wat vond je van het feit dat je het ene moment achter je eigen computer zat en het andere moment in de spreekkamer op de poli?"

TAAK CONTEXT TIJDENS HET AANBIEDEN VAN DE COMBI BEHANDELING

"Het online gedeelte van de behandeling is behoorlijk gestructureerd, terwijl de afspraken op de poli wat meer vrijheid kennen. Hoe kijkt u hier als counselor tegen aan?"

"Wat vond u vooral prettig aan de vrijheid / vaste structuur?"

Evtl. doorvragen naar afleiding door andere taken bij het online gedeelte.

"Hoe druk bent u geweest met die combi behandeling?"

"Hoe druk bent u geweest met andere taken tijdens de combi behandeling?"

TECHNISCHE EN INFORMATIEVE CONTEXT TIJDENS HET VOLGEN VAN DE COMBI BEHANDELING

"Wat vind u van de voorlichting die u hebt gekregen over de combi behandeling?"

"Had de computer die tot uw beschikking stond invloed op uw ervaring met de behandeling?"

EVALUATIE

Themalijst:

Gebruiksvriendelijkheid	❑
Gebruiksvriendelijkheid "online"	❑
Gebruiksvriendelijkheid "face-to-face"	❑
Verbeterpunten gebruiksvriendelijkheid	❑
Vanzelfsprekendheid	❑
Vanzelfsprekendheid "online"	❑
Vanzelfsprekendheid "face-to-face"	❑
Verbeterpunten vanzelfsprekendheid	❑
Toegankelijkheid	❑
Toegankelijkheid "online"	❑
Toegankelijkheid "face-to-face"	❑
Verbeterpunten toegankelijkheid	❑
Tevredenheid "algemeen"	❑
Tevredenheid "online"	❑
Tevredenheid "face-to-face"	❑
Verbeterpunten tevredenheid	❑
Verdeling face-to-face en online gedeelte	❑
Voordelen/Nadelen	❑

GEBRUIKSVRIENDELIJKHEID VAN DE COMBI BEHANDELING

"We willen het graag hebben over de gebruiksvriendelijkheid van de gecombineerde behandeling, denkt u hierbij aan vanzelfsprekendheid, het gemak in gebruik en toegankelijkheid."

"Kunt u vertellen wat uw ervaringen zijn met de gebruiksvriendelijkheid?"

"Kunt u hier voorbeelden van geven?"

"Waar denkt u dat er <u>verbeteringen</u> kunnen worden aangebracht in het programma?"

De volgende vragen alleen stellen als de respondent geen toelichting geeft op gebruiksvriendelijkheid van het online gedeelte van de combi behandeling.

"Hoe gebruiksvriendelijk vond u het online gedeelte van de combi behandeling?"

"Kunt u hier voorbeelden van geven?"

"Waar denkt u dat er verbeteringen kunnen worden aangebracht in het online deel van de combi behandeling?"

De volgende vragen alleen stellen als de respondent geen toelichting geeft op gebruiksvriendelijkheid van het face-to-face gedeelte van de combi behandeling.

"Hoe gebruiksvriendelijk vond u het face-to-face gedeelte van de combi behandeling?"

"Kunt u hier voorbeelden van geven?"

"Waar denkt u dat er verbeteringen kunnen worden aangebracht in het face-to-face deel van de combi behandeling?"

De volgende vragen alleen stellen als de respondent geen toelichting geeft op vanzelfsprekendheid van het online gedeelte van de combi behandeling.

"Wat is uw ervaring met betrekking tot vanzelfsprekendheid in het online gedeelte van de combi behandeling?"

"Kunt u ons hier wat meer over uw ervaringen vertellen?"

"Kunt u hier voorbeelden van geven?"

"Waar denkt u dat er verbeteringen kunnen worden aangebracht in de combi behandeling?"

De volgende vier vragen alleen stellen als de respondent geen toelichting geeft op vanzelfsprekendheid van het face-to-face gedeelte van de combi behandeling.

"Wat is uw ervaring met betrekking tot vanzelfsprekendheid in het face-to-face gedeelte van de combibehandeling?"

"Kunt u ons hier wat meer over uw ervaringen vertellen?"

"Kunt u hier voorbeelden van geven?"

"Waar denkt u dat er verbeteringen kunnen worden aangebracht in de combi behandeling?"

De volgende vier vragen alleen stellen als de respondent geen toelichting geeft op toegankelijkheid van het online gedeelte van de combi behandeling.

"Wat is uw ervaring met betrekking tot toegankelijkheid in het online gedeelte van de combi behandeling?"

"Kunt u ons hier wat meer over uw ervaringen vertellen?"

"Kunt u hier voorbeelden van geven?"

"Waar denkt u dat er verbeteringen kunnen worden aangebracht in de combi behandeling?"

De volgende vier vragen alleen stellen als de respondent geen toelichting geeft op toegankelijkheid van het face-to-face gedeelte van de combi behandeling.

"Wat is uw ervaring met betrekking tot toegankelijkheid in het face-to-face gedeelte van de combi behandeling?"

"Kunt u ons hier wat meer over uw ervaringen vertellen?"

"Kunt u hier voorbeelden van geven?"

"Waar denkt u dat er verbeteringen kunnen worden aangebracht in de combi behandeling?"

Tevredenheid met de combi behandeling

"Hoe is uw algemene tevredenheid over de combi behandeling?"

"Over welke onderdelen bent u minder of meer tevreden?"

"Hoe zouden we hierin verbeteringen kunnen aanbrengen volgens u?"

De volgende vier vragen alleen stellen als de respondent geen toelichting geeft op het online gedeelte van de combi behandeling.

"Hoe is uw tevredenheid over het online gedeelte van de combi behandeling?"

"Over welke onderdelen bent u het meest en minst tevreden?"

"Hoe zouden we hierin verbeteringen kunnen aanbrengen volgens u?"

„Als u mocht kiezen waar zou u dan zelf als eerste verandering in aan willen brengen? Waarom?

De volgende vragen alleen stellen als de respondent geen toelichting geeft op het face-to-face gedeelte van de combi behandeling.

"Hoe is uw tevredenheid over het face-to-face gedeelte van de combi behandeling?"

"Kunt u een voorbeeld geven van een onderdeel waar u minder tevreden over bent?"

"Hoe zouden we hierin verbeteringen kunnen aanbrengen volgens u?"

„Als u mocht kiezen waar zou u dan zelf als eerste verandering in aan willen brengen? Waarom?

"Kunt u een voorbeeld geven van een onderdeel waar u meest tevreden over bent?"

Verdeling face-to-face en online gedeeltes

"Wat vindt u van de verdeling face-to-face behandelingen en online sessies?"

"Wat vond u van de tijdsverdeling?"

VOORDELEN/NADELEN

"Wat zijn voor u de belangrijkste voordelen van deze combi behandeling?

“Wat zijn voor u de belangrijkste nadelen van deze combi behandeling?"

AFSLUITING

Themalijst:

Eigen toevoeging van de patiënt	❑
Omschrijving van de behandeling	❑

EIGEN TOEVOEGING VAN DE PATIËNT

„We zijn nu met onze vragenlijst eigenlijk klaar. Is er nog iets wat uw graag wilt zeggen of toevoegen waar wij het niet over hebben gehad?“

OMSCHRIJVING COMBI BEHANDELING

“Dan wil ik u ter afsluiting graag nog een korte vragen stellen die niet zozeer over uw ervaringen gaan, maar wat meer praktisch van aard zijn.”

„We hebben het telkens over de combibehandeling, maar merken dat het soms lastig is om de behandeling goed te omschrijven. Heeft u hiervoor een tip of suggestie?

Doorvragen met b.v.

“Als u de combi behandeling in <u>3 woorden</u> mag beschrijven, welke 3 woorden zouden dat dan zijn?“

AFSLUITING

- Check aan de hand van de topiclijsten of je alle vragen hebt gesteld en of je overal antwoord op hebt gekregen.
- Stel eventueel nog aanvullende vragen om de antwoorden compleet te maken.
- Kondig het einde van het gesprek aan.
- Geef de geïnterviewde de gelegenheid om vragen te stellen of om aanvullingen/ toelichtingen te geven
- Geef kort aan hoe de procedure van verwerking eruit ziet.
- Vraag de respondent om feedback over het interview: wat vond hij/zij van het interview?
- Bedank de respondent voor zijn/haar medewerking.

RAPPORT VAN DE INTERVIEWER

Onderstaande vragen graag meteen na afloop van het interview beantwoorden

1) Waren er buiten jezelf en de respondent nog andere personen aanwezig die de vragen en antwoorden konden horen?

- ☐ Niemand aanwezig (ga door naar vraag 3)
- ☐ Anderen aanwezig

2) Is de aanwezigheid van anderen volgens jou van invloed geweest op wijze waarop respondent de vragen heeft beantwoord?

- ☐ Nee/waarschijnlijk niet
- ☐ Ja/waarschijnlijk wel, namelijk

……
……

3) Eventuele opvallende zaken voor/tijdens/na het interview

……
……

……
……

……
……

……
……
