# Supplementary material for: Patients’ User Experience of a Blended Face-to-Face and Web-Based Smoking Cessation Treatment: Qualitative Study
Source: JMIR Form Res. 2020 Jun 3;4(6):e14550. doi: 10.2196/14550 (PMC7301265; doi:10.2196/14550)
Supplement: Multimedia Appendix 3 [file formative_v4i6e14550_app3.pdf]

Multimedia Appendix 2. Codes, code description, and intercoder agreement per semantic domain

Semantic Domain	Code	Description	Krippendorff's c-α-binary
User Experience	ux bsct	User experience with the blended treatment - Phrases in which the patient describes an experience during the treatment which he/she also combines with an evaluation and/or emotion (e.g. "good", "bad", "frustrating") Only the phrases that refer to the blended treatment, Not the ones referring only to F2F, or WEB or others (episodic and/or cumulative UX)	0.676
	ux f2f	User experience with the F2F parts of BSCT - As ux bsct, but only referring to the F2F parts of BSCT	
	ux web	User experience with the WEB parts of BSCT - As ux bsct, but only referring to the WEB parts of BSCT	
	ux others	User experience that cannot be related to other UX Codes - As above, but only if not referring to the other three UX codes	
System	motivation to follow bsct	Motivation to continue the treatment - The motivation to adhere to the treatment which are closely related to properties of the treatment itself, e.g. supporting elements of the treatment,	0.650
	presence of counselor online	What the patient thinks about the counselor while he/she is in the web-based part of the treatment - Description and ideas about what the patient thinks about the counselor while he/she is using the Web-based parts of the treatment, e.g. "having the counselor in mind while using the tools" or "feels like filling in a tax declaration"	
User	earlier treatment experience	Prior experience in F2F and WEB treatment/counselling - This is about treatment/counseling IN GENERAL, for prior experience in smoking cessation use code „smoking cessation experience. All experience in BSCT comparable treatment including treatment and counseling in health- or behavior related topic in an f2f, Web-based and/or group setting.	0.726
	expectation towards bsct	Expectation at treatment start - What patients expected when they were informed to be included in BSCT (anticipated UX)	
	health status	Additional information about health and mental status - Additional information about health and mental status which is not closely related to the motivation (use Motivation quitting OR Motivation BSCT)	
	it experience	Experience in using digital technology for communication - How the patient describes himself/herself in the use of computers, tablets, mobiles etc. for communication	
	mood during smoking cessation	Moods/emotions related to quitting - Description of moods and emotions related to quitting smoking. (only if not explicitly related to BSCT - use UX-codes for these)	
	motivation for smoking cessation	Motivation to quit or to stay abstinent - The motivation to quit and/or to stay abstinent which are not closely related to aspects of the treatment, but more related to e.g. smoking, health, family.	
	smoking cessation experience	Experience in smoking cessation treatment - Earlier experience in smoking cessation AND current experience in smoking cessation which is not related to the current treatment	
Context	physical context	Information about the physical context - Additional information about the physical context (e.g. housing situation, travel time) which is not closely related to the motivation (use Motivation quitting OR Motivation BSCT)	0.780
	social context	Information about the social context - Additional information about the social context (e.g. partner, friends, family) which is not closely related to the motivation (use Motivation quitting OR Motivation BSCT)	
	task context	What patients think about the structure and freedom during treatment - If not directly related to Motivation or UX	
	technical context	Information about the technological context - Information about the technological context such as computers, laptops, internet connection. Use only if phrase is not referring to Motivation or UX	
Structural	improvements	Ideas and recommendations for further improvement of BSCT	0.928
	intake	How the patients were informed before start of the treatment - Phrase of patients in which they describe the quality of the intake procedure and which are helpful for improvement of the treatment (do not mix up with Expectation)	
	name of the treatment	Phrases of the patient in which he/she calls or describes BSCT in his/her own words in a "striking" expression	
	patient characteristics matching bsct	Characteristics of users that increase matching between treatment and user - Phrases of patients in which he/she describes characteristics of users that best match with BSCT, e.g. "you need to be computer-literate"	
	procedures	Additional information about the procedures of the treatment - Phrase of patients in which they describe the procedures of the treatment and which are helpful for improvement of the treatment, but which are not closely related to the UX	
Miscellaneous	advantages	Phrases in which the patient summarizes advantages of BSCT	0.744
	disadvantages	Phrases in which the patient summarizes disadvantages of BSCT	
	preference of treatment mode	Which kind of treatment the patient would prefer next time or advise to others - Phrases in which the patients evaluate the modes of the treatment by telling which mode of treatment he/she would go for next time or he/she would advise to others	
	miscellaneous		
