# Supplementary material for: Patients’ User Experience of a Blended Face-to-Face and Web-Based Smoking Cessation Treatment: Qualitative Study
Source: JMIR Form Res. 2020 Jun 3;4(6):e14550. doi: 10.2196/14550 (PMC7301265; doi:10.2196/14550)
Supplement: Multimedia Appendix 4 [file formative_v4i6e14550_app4.pdf]

Multimedia Appendix 3. Selective sample & key elements

(1) Patients' standards and expectations

Randomization number	#10	#12	#14	#25	#27	#34	#53	#75	#106	#509
Standard (F2F treatment)		F2F is ideal<br>F2F adapts to your competences	F2F is harder if you do not like the counselor<br>Written information has less impact than F2F		No earlier F2F treatment<br>Following treatment if it is paid by the insurance company	Uses mindfulness app<br>Earlier group therapy (heart revalidation), which did not meet his needs	No earlier F2F treatment<br>Experienced in mindfulness myocardial infarction<br>You just have to do the things	Human touch is important		No earlier F2F treatment
Standard (IT)	Computer is not my way<br>I am able to use computers<br>I don't like online	I am not a computer freak<br>It is too easy to lie and you have to be serious to do it.<br>You have to be a computer freak and not been interested in personal touch,<br>able to process written information<br>Correctness: You have to be 100% correct for web. Age: Young people are skilled with computers, but not interested in health. Older people do not like and do not trust computers.	don't' want a computer in the living room	I followed all IT developments<br>I use the laptop less since I have a tablet<br>Computer is a tool<br>I don't like to be accessible 24/7<br>I don't like messenger apps<br>Neither a forerunner nor a left behind<br>Written has more impact<br>it is a problem of generation<br>You do not remember information because it is always accessible<br>I use books less<br>Tablet is playing, Laptop is serious (=smoking cessation)<br>I can't imagine spending much money (>50€/month) on a mobile phone<br>Online information is not as important as written op paper	Prefers mobile (Facebook, WhatsApp, Talking) instead of laptop<br>I do not check my emails regularly<br>I only check my emails after being reminded<br>I prefer mobile over PC	Used to computers because of work.<br>I personalize my mobile<br>I am a technician and used to computers<br>is easier to do sloppy<br>It is easy to cheat online<br>Web you can do sloppy<br>Conscientiousness	Used to computers<br>Computer mediated communication lead to misunderstandings<br>I prefer F2F communication for relevant stuff	Computer is a tool<br>Everyone knows computers are relevant and may be an addition	I go for everything mobile<br>I prefer mobile over PC<br>I use standard apps<br>Today, children grow up with mobiles<br>Use google for search	Use computers on a „needs" basis, for getting things done<br>Computer is a tool<br>I am not interested in high end smartphones<br>Facebook is nonsense<br>Texting can be useful sometimes<br>Use google for search<br>I do not trust internet information
Standard (Web-based treatment)				Online is useful treatment as "a big stick" („stock achter de deur")	No earlier Web treatment	Uses mindfulness app<br>I have the Stoptober app Web	No earlier Web treatment			No earlier Web treatment
Standard (quitting)	Pleasure not to smoke<br>Stress causes relapse	Stopping you have to do for yourself	You must have the will<br>Treatment only makes sense if you have the will to stop<br>With help stopping will be easier		Stress causes relapse<br>It is more mental than physical problem<br>You have to be strong<br>Weight gain impacts smoking cessation<br>It's up on you	Earlier unsuccessful quitting attempts (without treatment)<br>Smoking is bad<br>Stress at work triggers smoking	Earlier unsuccessful quitting attempts (without treatment)<br>With help stopping will be easier<br>Examples and warnings	circumstances cause relapse<br>Stress causes relapse<br>other medication causes relapse	Earlier unsuccessful stop smoking therapy with medication only	
Expectation (treatment)	Counselors will be "a big stick"	Web will not work for me,	Wait and see because it is new<br>Sounds interesting	You simply have to do it, even if it is not fun<br>No clear idea<br>I did it because I thought it was obligatory<br>Thinking it was obligatory did not increase motivation for the treatment	Neutral, no expectations, no clear picture<br>expected long examination	to make a new step<br>to get more grip on smoking cessation<br>No clear idea<br>Web will be comfortable<br>to get told how to stop and how it will be supported	support in smoking cessation<br>open	Open, no clear expectation or picture, hope to reduce smoking	Neutral, no expectations	Good expectations, because you will make it

(2) Apparent character of BSCT - pragmatic attributes

Randomization number	#10	#12	#14	#25	#27	#34	#53	#75	#106	#509
Usability (BSCT)	Intake was good<br>Procedures are fine<br>Intervals between session are too long<br>Treatment is picked up well<br>BSCT is easy-to use<br>F2F and Web are equally time demanding	Long waiting list	Everything was explained well<br>that was all quite logical<br>Long waiting list<br>Long time in the waiting area<br>Not clear where to turn to outside the office hours<br>Everything was clear<br>BSCT parts connect to each other<br>You can ask F2F if you have questions about Web	Not everything was explained in detail<br>I was surprised about the order of sessions<br>Expected intervals between F2F to be shorter	Treatment is picked up well<br>I was called to be reminded of my sessions	Everyone is kind<br>Less travel<br>No parking problems<br>Les time consuming	Everything was clear<br>BSCT is easy-to-use<br>BSCT parts connect to each other<br>Treatment is picked up well Intervals between sessions are fine	I was introduced well to the treatment<br>No problems<br>BSCT parts connect to each other<br>F2F and Web support	BSCT parts connect to each other	No problems
Usability (F2F)	Counselor does not have enough time<br>Disadvantage: Travelling to hospital	Counselor does not have enough time	Counselor does not have enough time<br>F2F is slow and time consuming<br>Less travel	F2F is easy to use	F2F is easier than Web<br>F2F is slow and time consuming					
Usability (Web)	Online is difficult		Online is easy to understand<br>Online is easy to us<br>Web you do not get immediate response<br>Online is a lot of repetition	Online is difficult<br>Online is NOT easy to use<br>Online is efficient (saves time)<br>If online is important I will print it on paper	Online is a lot of repetition<br>Online is easy to use<br>I did not receive online assignments<br>Web you have to log in each time and you have to remember your password<br>Web you have to do very often<br>Online information that has to be filled in should be link to earlier data, so that you do not fill in twice<br>Easier Login so you do not have to remember your password	Online is not easy to use<br>Emails/phone calls raise awareness<br>Filing in forms online is handy<br>Web is more practical than<br>F2F	Online is a lot of repetition Online is easy to use	Online is easy to use<br>I enjoyed being notified automatically about new Web content	I did the smoking registration on paper before doing it online<br>Keeping the smoking diary on paper is simpler than online<br>Online is easy to use<br>Online is too time demanding<br>During work I kept record of smoking on paper, which is double work	Online is too time demanding<br>I did the smoking registration on paper before doing it online<br>Online is too time demanding<br>Online is easy to use<br>Online is a lot of repetition
Utility (BSCT)				Setting goals is important<br>Changing habits is important	BSCT offers support<br>Solution-focused therapy		All BSCT parts were helpful – some more, some less<br>BSCT did match my quitting process	Web only would not have offered what I needed<br>It is good talk about it later	All has been discussed<br>There is progress	
Utility (F2F)	Counselors stimulated<br><br>F2f stimulates more than Web	F2F works for me only<br>Counselor does not offer enough support<br>Counselor does not motivate<br>Counselor does not reinforce	It was offered to ask questions every time<br>Counselors stimulated quitting<br>All has been discussed<br>Counselor offers support<br>Counselors explained everything very well<br>Counselors explained everything very well<br>F2F you get direct answers<br>F2F fast answers on questions<br>F2F it is easier to ask questions<br>Medication was done in consultation with the counselor<br>Following treatment after stopping is not necessary but OK	Counselors reinforce<br>Counselor offers support<br>F2F you get written information	Counselor does not offer enough support<br>Counselors asks too much questions<br>Counselor does not offer enough support	F2f you can't hide<br>Counselor does not offer enough support<br>Counselor did not discuss alternatives	Counselors reinforce<br>Counselor offers support<br>Counselors shares good metaphors	F2F offers flexibility<br>It is good to talk after a fail	F2F+mediaction is better than medication only	I talked to the counselor about all of my problems<br>More personal contact
Utility (Web)	Multiple choice questions are annoying		Online you do not get immediate answers to questions<br>Information available online<br>Online did not match my quitting process<br>Computer does not answer		Online does not match my quitting process<br>Information available online	Information available online after finalizing treatment	Online offers support in difficult moments<br>NRT offers more support than Web in difficult moments<br>Online offers tips	Online does not work for me	I was reinforced to work online<br>Online offers too much information<br>App<br>short reminders (several times a day) to track smoking<br>shorter texts	Online registration is so time demanding that it prevents smoking<br>Reporting via Web Weekly report about own smoke behavior (not several time a day tracking)

(2) Apparent character of BSCT - hedonic attributes

Randomization number	#10	#12	#14	#25	#27	#34	#53	#75	#106	#509
Stimulation (BSCT)	Web was not motivating	I was reinforced to do Web	BSCT stimulated quitting<br>BSCT is interesting<br>BSCT is different from F2F only	You just have to do the things<br>Dealing with tempters was not new	Not digging so deep always the same questions is demotivating<br>Digging too deep is demotivating<br>contradictory goals (quitting, weight reduction) demotivate	BSCT did not offer new things<br>More positive reinforcement Because it made me think			Because it made me think	digging deeper
Stimulation (F2F)		Counselor has no impact<br>Counselor does not care whether I quit Smoking<br>CO measurement stimulates quitting<br>Discussing costs of smoking stimulates quitting	Counselor has no impact	Counselors stimulate	Counselor „digs too deep"<br>Counselor has no impact		Counselors stimulated quitting<br>Counselors reinforce<br>CO measurement stimulates quitting		Counselor reinforced<br>Counselor made me think about the risks of smoking	Counselor has no impact
Stimulation (Web)	Online is not interesting		Online won't get through to me<br>Online is not interesting<br>Online offers possibility to look back	Online exchange with the counselor does not affect extraordinary<br>Online won't get through to me.		I was reinforced to work online<br>Online won't get through to me	Online won't get through to me.<br>Online stimulates to think	Online offers possibility to look back<br>First it is technical<br>It broadens your awareness	Computer use demotivates	Online motivation decreases over time<br>I was reinforced to work online<br>Online exchange with the counselor does not affect extraordinary<br>Online motivation decreases over time
Identification (BSCT)			You do not know who has written the content	I had to make the best out of it<br>I do not do it for no one<br>In F2F session it became clear that the counselor reads the Web content	Lose your rhythms<br>Fells treated like a child			Able to work based on reading and writing<br>Perseverance		I have contact with here<br>They know something about me
Identification (F2F)	I like the counselors ("Talking to the ladies")						F2f feels more familiar		I like the F2F sessions the most	
Identification (Web)	Online is not my style<br>Too stupid for IT Age<br>IT skills<br>I don't like online	Online is not my style Computers does not talk to you, you do not get the feeling that there is a human being on the other side	Computer does not talk back	I don't benefit from Web because it is not my style<br>Prefer on paper		You know there is someone behind it	I had the idea that it is used on the other side	Online does not give the opportunity to make it more personal<br>Web I do for myself<br>Self-control to follow Web<br>I know why I do Web. I understand the process	I don't feel like it much<br>I don't like online	
Evocation	Online is like filling in tax forms		Online feels like bookkeeping		Computer is like paper<br>Audio information (listening to text	An app would be better than Web<br>Online feels like bookkeeping<br>short reinforcements via WhatsApp, Email in-between sessions<br>Video instructions<br>helpdesk, chat support<br>short instructions	Online feels like bookkeeping Online is like a manual		Like handling a machine, because you are not sitting opposite to each other	

(3) Situation

Randomization number	#10	#12	#14	#25	#27	#34	#53	#75	#106	#509
Technical	Laptop broke, got a new iPad instead<br>Stopped Web because of moving to iPad<br>I received content on the iPad<br>iPad is more comfortable than Laptop		Technology had no problems<br>Web did not work on iPad<br>Starting up Laptop takes time	Laptop broke, got a new iPad instead<br>Tablet is always on, Laptop not<br>I choose to use tablet for other tasks than laptop		Web did not work on iPad<br>Tablet is always on, Laptop not<br>Starting up Laptop takes time		Unfortunately, some content does not run on tablets	had to start up the laptop which costs time	
Task					sometimes stress, sometimes relaxed				not enough time because of family tasks	not enough time because of other tasks<br>Too much to do to stop smoking
Physical (Hosptial)				I live close to the hospital	My treatment was at the old building, not a nice place	My treatment was at the old building, not a nice place				
Physical (Home)	hobby room upstairs<br>nice place		Upstairs<br>In summer it is hot upstairs<br>You have to go upstairs to the computer	I have my own office at home			Laptop at the dining table with wife and children around me	Comfortable at the computer<br>Some session via phone	in the kitchen	PC in my office at home<br>with Laptop lying on the bed in the sleeping room
Social (Family)	My partner stimulated and offered incentives<br>My partner is accompanying me, quite sociable		My partner deals with my moods during quitting<br>My son also quit<br>Nearly no one in our family smokes	I did not tell much about BSCT<br>I live alone<br>Everyone supported me	We have nine children<br>My children supported me<br>My husband stills smokes, did not support<br>Partner did not support, questions web sessions<br>Partner brakes agreement to smoke outside only		Life event: Myocardial infarction<br>Family (daughter) is anxiously because of health<br>Brother died of myocardial infarction<br>Family is worried<br>Everyone supports and compliments<br>Partner gives feedback on better health conditions, compliments, gives incentives<br>Children are positive about quitting		Family motivates stopping	My family relies on me<br>My partner does no smoke<br>My partner stimulates
Social (Friends)	I stimulate others to quit smoking<br>None of my friends smokes		No one smokes inside				Colleagues also have positive experiences with Cessation treatment			
Social (Society)		Because of smoking you do not feel ashamed								

(4) Consequences

Randomization number	#10	#12	#14	#25	#27	#34	#53	#75	#106	#509
Appeal (BSCT)			Mix of talking and reading<br>Sometimes F2F is better – sometimes Web is better<br>BSCT is good idea	Good<br>I do not do it for no one	BSCT is good<br><br>Champix was good	F2f and Web are quite different	BSCT is good<br>Web only would not have been so easy<br>Web is an extension of F2F	BSCT is good<br>BSCT offers variety<br>I had to go to the hospital less often	BSCT is good<br>Shared information both F2F and Web is fine	
Appeal (F2F)		F2F touches your heart<br>100% personal in all facets			Counselor is nice<br>F2F session are "whiny"	F2F talks are most important at treatment start<br>Counselor has a "stiff posture"<br>Counselor is pedantic<br>Online is cumbersome<br>Counselor is annoying me	F2F talks are most important		F2F is finer than Web	F2F talks were fine
Appeal (Web)	Online is dead<br>Online is nonsense	Web is nonsense	Online yielded nothing	Not Web only<br>Online is a serious matter	Online is a lot<br>Online is boring<br>Online is tiring	Online is boring<br>Comfortable at home	Online yielded nothing	Online yielded nothing<br>Online is nice	Online is cumbersome (compared to apps)	Online is a lot<br>Online yielded nothing
Emotion (BSCT)	I am satisfied with BSCT	Feeling abandoned and left alone		I am not satisfied with BSCT<br>F2F and Web stimulated the same moods	I am satisfied with BSCT<br>Inconsistent/Contradictory quitting smoking and weight reduction	I am not satisfied with BSCT		Needed to relax physically tension	I am thankful	
Emotion (F2F)					F2F sessions were in a good mood	guilty because I did not stick to appointments	F2F session were in a good mood			
Emotion (Web)					Online is unpleasant	Keeping the online smoking diary makes me nervous				
Emotion (quitting)	it is hard<br>I had to fight while being somehow depressed<br>Grumpy<br>Rotten mood<br>Indifferent if I am over the hump<br>ups and downs<br>I had to fight<br>Craving while being stressed		No craving<br>100% lucky<br>it was easy<br>not grumpy<br>good mood<br>I had to fight for some days, then it was easy<br>I did it myself<br>has been very strange and that's why I say that I have never experienced that it went so easy	Changing mood<br>Weak and guilty because I did not stop	Inconsistent<br>Disgust<br>Fight with yourself<br>Relapse felt dirty	Proud<br>it is hard<br>need for support<br>annoyed about myself	Stress<br>comfortable<br>not grumpy<br>feeling good because of success	Stress		stress
Behaviour (treatment)	I did not do online.<br>Online I did sloppy	Non-adherend,	I did everything online<br>Following treatment to do the counselors/researchers a favor<br>Could have stopped after 4 times, because I was sure<br>do not need it in the future<br>adherent/	I did both F2f and Web<br>I did not do online<br>Online I did sloppy	I forgot about some of my session<br>I used both "on paper" and Web<br>Online I did sloppy	Online I did sloppy tracking my smoking increased smoking	I did both F2f and Web	Adherent,	Online I did sloppy	
Behavior (quitting)		non-quitter	quitter				I told myself „Never again"<br>I told myself „enough" (basta)<br>I saved money for the holidays with my family	reduced smoking	No problems because I had medication (Champix)<br>I threw away my last shag	Pain in my leg made me smoke<br>I smoke less<br>You smoke less if you have too much work<br>Online registration is so time consuming that it prevents smoking<br>I do not smoke for hours in the sleeping room or if I am quite busy
